# Differential gene expression in *Aspergillus fumigatus* induced by human platelets *in vitro*

**DOI:** 10.1016/j.ijmm.2015.01.002

**Published:** 2015-05

**Authors:** Susanne Perkhofer, Christoph Zenzmaier, Emilie Frealle, Michael Blatzer, Hubert Hackl, Bettina Sartori, Cornelia Lass-Flörl

**Affiliations:** a University of Applied Sciences Tyrol, 6020 Innsbruck, Austria; b Division of Hygiene and Medical Microbiology, Innsbruck Medical University, 6020 Innsbruck, Austria; c Center for Infection and Immunity of Lille (CIIL), Institut Pasteur de Lille, Biology and Diversity of Emerging Eukaryotic Pathogens (BDEEP), INSERM U1019, CNRS UMR 8204, Univ. Lille Nord de France, Lille, France; d Division of Bioinformatics, Biocenter, Innsbruck Medical University, 6020 Innsbruck, Austria

**Keywords:** *Aspergillus fumigatus*, Aspergillosis, Platelets, Microarray, Thrombocytes, Transcriptome

## Abstract

Invasive aspergillosis is characterized by vascular invasion and thrombosis. In order to determine the antifungal activity of human platelets, hyphal elongation and metabolic activity of a clinical *A. fumigatus* isolate were measured. Genome-wide identification of differentially expressed genes in *A. fumigatus* was performed after exposure to platelets for 15, 30, 60 and 180 min. Data were analyzed by gene ontology annotation as well as functional categories (FunCat) and KEGG enrichment analyses.

Platelets attenuated hyphal elongation and viability of *A. fumigatus* and in total 584 differentially expressed genes were identified, many of which were associated with regulation of biological processes, stress response, transport and metabolism. FunCat and KEGG enrichment analyses showed stress response and metabolic adaptation to be increased in response to platelets. Our findings demonstrate that *A. fumigatus* displayed a specific transcriptional response when exposed to platelets, thus reflecting their antifungal activities.

## Introduction

Invasive aspergillosis is increasingly recognized in immunocompromised hosts (Maschmeyer et al., 2007; Taubitz et al., 2007) and patients with prolonged and deep granulocytopenia following chemotherapy for hemato-oncologic disorders or allogenic bone marrow transplant recipients are particular at risk. The crude mortality from invasive aspergillosis lies around 85% and decreases to around 50% when treated (Denning, 1998). *Aspergillus fumigatus* is the most prominent pathogen in the *Aspergillus* genus, accounting for more than 90% of human infections (Maschmeyer et al., 2007; Taubitz et al., 2007).

In immunocompromised patients the immune system fails to eliminate conidia, which can then germinate and cause infection (Maschmeyer et al., 2007). A key process in invasive aspergillosis is angioinvasion of hyphae (Hogan et al., 1996; Kamai et al., 2006), which allows hyphae to enter the bloodstream, to invade blood vessels and the deep tissues. Subsequently, interaction between *Aspergillus* and human platelets results in thrombosis and tissue infarction (Kamai et al., 2006).

Innate, humoral and cell-mediated immunity are involved in host defence against fungi. Although the contribution of each appears to be site-specific, polymorphonuclear leukocytes and macrophages dominate in protecting against aspergillosis (Nucci and Marr, 2005; Schaffner et al., 1982). Platelets also play key and multifaceted roles in antibacterial host defence and exhibit features characteristic of classic cell-mediated immune effector cells (Fitzgerald et al., 2006; Klinger and Jelkmann, 2002; Page, 1989). Only few data on the antifungal role of platelets against *Aspergillus* are available (Christin et al., 1998; Perkhofer et al., 2008)*.* It is known that in the presence of *A. fumigatus* platelets express the glycoprotein CD63 and supplement polymorphic neutrophils in defence against aspergilli (Christin et al., 1998). We observed that serotonin acts fungicidal against *Aspergillus* and attenuates fungal virulence *in vitro* (Perkhofer et al., 2007; Perkhofer et al., 2008). Serotonin is stored in the dense granules of platelets at a concentration of 65 mM and is immediately released after contact with *Aspergillus* spp. (Perkhofer et al., 2008). Germination and hyphal elongation of *Aspergillus* were significantly affected when exposed to platelets (Perkhofer et al., 2008). Additionally, the polysaccharide galactomannan, an important structural component of the cell wall of *Aspergillus* spp., which is released by growing hyphae, was significantly reduced under platelet treatment (Perkhofer et al., 2008).

Reports on the transcriptional response of *Aspergillus* spp. exposed to human cells are limited. Genes differentially expressed in *A. fumigatus* in response to neutrophils, monocyte-derived dendritic cells and airway epithelial cells have been investigated previously (Morton et al., 2011; Sugui et al., 2008; Oosthuizen et al., 2011). Using genome-wide microarray, the present study aimed to identify genes differentially expressed in *A. fumigatus* following exposure to human platelets to gain better understanding of their contribution to antifugal host defence.

## Materials and methods

### Ethic statement

This study was conducted according to the principles expressed in the Declaration of Helsinki. All healthy donors gave written informed consent to the use of residual platelet concentrates for research purposes. All residual platelet concentrates were de-identified and used anonymously.

### Fungal strain and growth conditions

*Aspergillus fumigatus* A22 was used in this study. The isolate was obtained from a patient suffering from invasive aspergillosis and stored in water at room temperature. Subcultures were grown on Sabouraud dextrose agar (SDA, Merck) and incubated at 37 °C for four days. The conidial suspension was harvested by flooding each colony with 2 ml H_2_O. Freshly harvested conidia were counted with a hemocytometer, and the final inoculum density was 1 × 10^5^ − 2.5 × 10^5^ colony forming units (cfu)/ml unless otherwise indicated. Viability and inoculum size were checked using cfu and for hyphal growth conidia were incubated in RPMI 1640 for 16 h at 37 °C shaking (150 rpm); > 90% formed hyphae under these conditions.

### Platelets

Platelets concentrates were provided from the local Department of Transfusion Medicine. Platelets were collected from regular healthy blood donors and prepared by thrombocyte apheresis with Amicus cell separator (Baxter). The platelet counts were 6 × 10^8^ cells/ml.

### Hyphal elongation

The morphology of conidia treated with platelets was examined assessing hyphal elongation using a platelet-to-conidia ratio of 100:1 for 18 h (Perkhofer et al., 2008). Platelets were lysed with ice-cold water and a micrometer was used for hyphal length measurement. The conidial inhibition rate was calculated from the percentage of conidia which did not germinate. Each sample was assessed in triplicate, measuring 50 conidia per sample.

### Determination of metabolic activity

Metabolic activity of hyphae was assessed using viability staining with FUN-1 (Molecular Probes). Therefore, conidia were incubated for 16 h at 37 °C to form hyphae prior to incubation with platelets at a platelet-to-conidia ratio of 100:1 for 5 h. Subsequently, hyphae were stained with FUN-1 at a concentration of 5 μM as described previously (Lass-Flörl et al., 2001). As control, *A. fumigatus* hyphae were incubated in the absence of platelets in medium containing 1.5 μg/ml amphotericin B, a concentration above the MIC (Lass-Flörl et al., 2008).

Additionally, hyphal damage caused by platelets was determined by the colorimetric 2,3-bis [2-methoxy-4-nitro-5-sulfophenyl] 2 H-tetrazolium-5-carboxynilide sodium salt (XTT) test assay plus 40 μg/ml coenzyme Q (2,3-dimethoxy-5-methyl-1,4-benzoquinone; Sigma-Aldrich), which is an established indicator of fungal cell damage (Roilides et al., 1995). Conidia were incubated for 16 h prior to addition of platelets for 30, 60 or 120 min. Platelets were lysed by washing three times with ice-cold H_2_O and then hyphae were incubated with XTT/coenzyme Q for 1 h at 37 °C. Subsequently, absorbance was determined at 450 nm using an ELISA plate reader (ASYS Hitech) and antifungal activity was quantified as percentage of hyphal damage, namely – (1 − *X*/*C*) × 100 where *X* is the optical density of test wells and *C* is the optical density of control wells with hyphae only. Three repetitions were performed in duplicate.

### RNA extraction

Conidia were incubated in RPMI 1640 for 16 h at 37 °C to form hyphae. Subsequently, platelets were added to hyphae for an incubation period of 15 min, 30 min, 1 h and 3 h at a platelet-to-conidia ratio of 100:1. Untreated fungi served as control. Frozen hyphae from un- and platelet-treated *A. fumigatus* cultures were ground in liquid nitrogen prior to RNA isolation using TRI Reagent (Sigma-Aldrich) according to manufacturerʼs instruction followed by phenol-chloroform-isoamylalcohol (PCI) extraction. RNA was purified using DNAse digestion (Qiagen) according to the manufacturerʼs protocol. The purity of RNA was measured by A260/280 and A260/230 ratios. Samples with ratios <1.8 for either ratio were not used for microarray analysis. Additionally, RNA quality was checked by Febit Holding GmbH (Germany) using an Agilent 2100 Bioanalyzer.

### Transcriptome profiling

RNA labeling for mRNA expression analysis and chip hybridization was performed by Febit Holding GmbH (Germany) using customized Geniom^®^ Biochips comprising 9500 elements (*A. fumigatus* transcripts).

Biochip signals were corrected for background and spatial effects and normalized using variance stabilizing normalization (VSN) (Huber et al., 2002). Changes in gene expression for each time point versus control were calculated as log2-ratio of the median at the respective time point and the median from the control samples. Significance was tested using moderated *t*-test (limma (Smyth, 2004)) and *p*-values were adjusted for multiple hypothesis testing using the Benjamini-Hochberg method (Benjamini and Hochberg, 1995). Genes were considered differentially expressed, if they were at least two-fold up- or down-regulated. Subsequently *K*-means clustering (*k* = 5) were performed on gene expression profiles differentially expressed (*p* < 0.05) in two or more time points and heatmaps were generated using Genesis (Sturn et al., 2002).

Further annotation was based on the *A. fumigatus* Af293 reference genome (http://www.cadre-genomes.org.uk/Aspergillus_fumigatus/Info/Index) and NCBI resources (http://www.ncbi.nlm.nih.gov/gene/). Genes differentially expressed at least at one time point were annotated to higher level parent GO Biological Process terms (GO Slim terms) using AmiGO 1.8 GO slimmer tool (http://amigo1.geneontology.org/cgi-bin/amigo/slimmer) with the pre-existing *Aspergillus* GO slim set. Different classification systems like FunCat (Functional catalog; Ruepp et al., 2004) and KEGG (Kanehisa and Goto, 2000) enrichment analyses for each time point were performed using FungiFun Version 0.5 (https://sbi.hki-jena.de/FungiFun/FungiFun.cgi; Priebe et al., 2011). Microarray data are deposited in the ArrayExpress database (www.ebi.ac.uk/arrayexpress) with the accession number E-MTAB-3024.

### Reverse transcribed qPCR validation of microarray data

Two-step RT-PCR was performed from total RNA using i-script reverse transcription supermix for RTqPCR and SsoFast EvaGreen Supermix (Bio-Rad). For cDNA synthesis 750 ng RNA was used as template, cDNA synthesis was performed according to the manufacturers recommendation. First strand cDNA was diluted 20× and 2 μl were used as template for PCR. Total volume of real time qPCR reactions came to 20 μl; SsoFast EVAGreen master mix, forward and reverse primer and cDNA. Cycling parameters were set up according to the recommendation of SsoFast EvaGreen Supermix (Bio-Rad). Non-template controls for each primer set were assayed to confirm no DNA contamination or primer dimer formation being present. Real time PCRs were performed in triplicates, and the expression levels of all genes of interest were normalized to ß–tubulin. The thermal cycling parameters consisted of an initial denaturation at 98 °C for 30 s followed by template amplification of 40 cycles of 98 °C for 5 s, 58 °C for 5 s in a CFX96 real time detection system (Bio-Rad). Fluorescence was measured during the annealing/extension step (58 °C) and a disassociation analysis (melting curve) was performed to confirm that a single amplified product was present. Data were analyzed using the 2^−ΔCT^ method (Livak and Schmittgen, 2001). For primer design sequences for *A. fumigatus* were retrieved from the Central *Aspergillus* REsource site (Mabey Gilsenan et al., 2012). Primers were designed to span exon intron boarders, amplicon lengths ranged between 103 and 135 bp with an annealing temperature of 58 °C. Gene identities, primer sequences and qPCR results compared to microarray data are given in supplemental Fig. 1.

### Statistical analysis

Data are presented as mean values, and error bars indicate ± S.E.M. Statistical analysis was performed using GraphPad Prism. For hyphal elongation statistically significant values relative to control are indicated by one-way ANOVA with Dunnettʼs multiple comparison test.

## Results

### Platelets attenuate hyphal elongation and metabolic activity of A. fumigatus *in vitro*

**Fig. 1:**
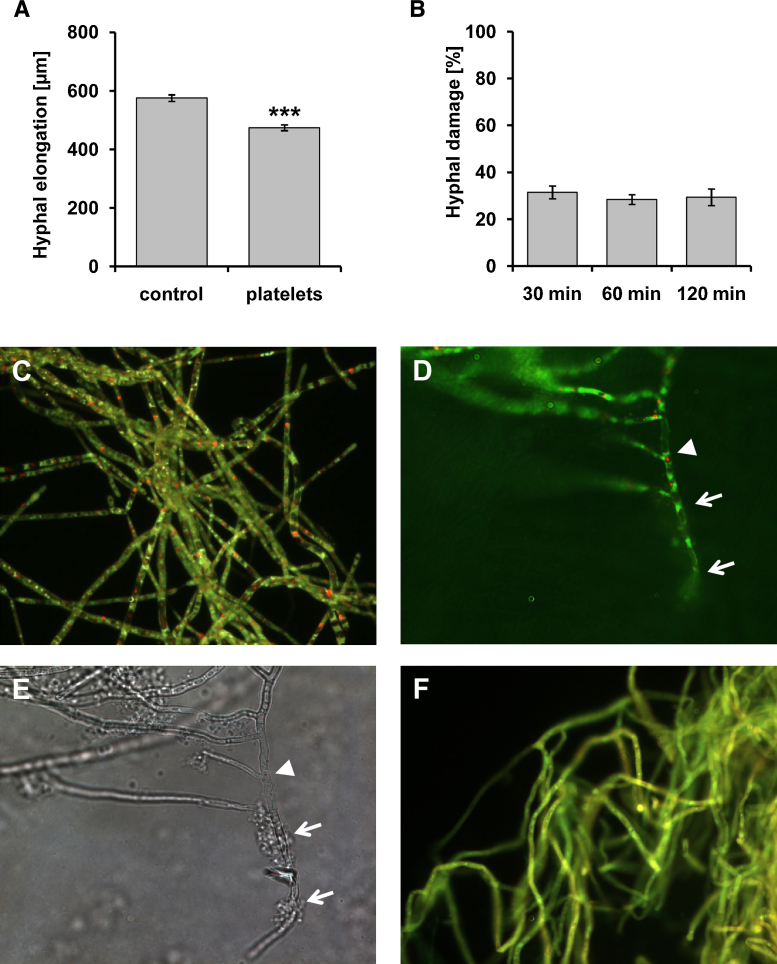
Platelets impact on hyphal elongation and metabolic activity of *A. fumigatus*. (A) *A. fumigatus* was incubated for 16 h in the absence or presence of platelets at a platelets to conidia ratio of 100:1 prior to determination of hyphal elongation. Bars represent mean ± SEM of three independent experiments. Significance versus control (0) treatment is indicated (^***^p < 0.001). (B) Conidia were incubated for 16 h to procude hyphae and platelets were added for the times indicated. Thereafter hyphal damage was analysed by XTT assay and untreated platelets served as control. Antifungal activity was calculated as percentage of hyphal damage and bars represent mean ± SEM of three independent experiments. (C) Viable untreated *A. fumigatus* is characterized by green fluorescent hyphae with clearly red fluorescent vacuole structures in FUN-1 staining. (D) Exposure to platelets impaired *A. fumigatus* viability as shown by green fluorescent hyphae and lack of red fluorescent vacuole structures at sites of hyphal-platelet contact identified by light microscopy (E; arrows). (F) Amphotericin B-treated *A. fumigatus* showed no remaining red fluorescent vacuole structures.

According to our previous findings (Perkhofer et al., 2008; Perkhofer et al., 2013) platelets significantly attenuated hyphal elongation of *A. fumigatus* (Fig. 1A). Additionally, metabolic activity of *A. fumigatus* hyphae was investigated. As determined by XTT assay, treatment with platelets reduced metabolic activity of *A. fumigatus* hyphae by 30% within 30 min and prolonged co-cultivation for up to 120 min did not significantly affect the extent of hyphal damage (Fig. 1B). Untreated fungus showed green fluorescent hyphae with clearly red fluorescent structures in vacuoles in FUN-1 stainings indicating metabolic activity (Fig. 1C), while treatment with platelets led to reductions in red fluorescent vacuolar structures at sites of hyphae-platelets contact (Fig. 1D and E, arrows); remaining red fluorescent structures display where platelets did not attach and aggregate (Fig. 1D and E, arrowhead). Controls treated with the fungicidal drug amphotericin B displayed no red fluorescent structures indicating dead cells (Fig. 1F).

### Genes differentially expressed in *Aspergillus fumigatus* upon exposure to human platelets

**Fig. 2:**
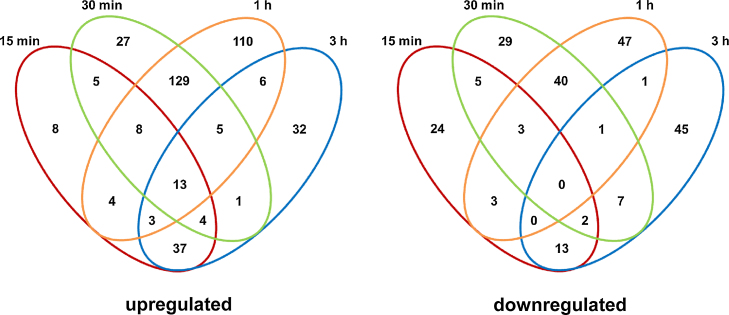
Genes differentially expressed in *Aspergillus fumigatus* upon exposure to human platelets. Venn diagrams showing genes that are at least two-fold up- or down-regulated in *A. fumigatus* after 15 min, 30 min, 1 h or 3 h of exposure to platelets. Genes significantly differentially expressed at least at one time point were included.

Since platelets significantly affected *A. fumigatus* growth and activity *in vitro*, genome-wide identification of differentially expressed genes in *A. fumigatus* was performed after platelets exposure for 15 min, 30 min, 1 h and 3 h. In total 584 genes were found differentially expressed (*p* < 0.05 at least at one time point), 220 of which were down-regulated and 392 up-regulated at least two-fold at one time point (supplemental Table 1, Fig. 2). Gene expression changes were most distinct after 1 h (371 genes, 93 genes down-regulated and 278 genes up-regulated) and 30 min (279 genes in total, 87 genes down- and 192 genes up-regulated), while less pronounced after 15 min (132 genes, 49 genes down- and 83 genes up-regulated) and 3 h (172 genes, with 71 down- and 101 up-regulated genes) (supplemental Table 2, Fig. 2). 199 genes were up- or down-regulated at both 30 min and 1 h, whereas less overlap occurred after 15 min and 30 min (40 genes), as well as after 1 h and 3 h (29 genes). Only 13 genes were found at least two-fold up-regulated at all four time points (Fig. 2). 90 genes were significantly (*p* < 0.05) differentially expressed at least at two time points. *K*-means clustering on gene expression profiles of these genes were performed and the resulting heatmap is shown as supplemental Fig. 2. To validate the changes in expression levels of the microarray experiments, 16 genes were selected for RT-qPCR analysis. Genes tested showed a similar expression pattern for both RT-qPCR and microarray (supplemental Fig. 1).

### Gene ontology classification of differentially expressed genes

**Fig. 3:**
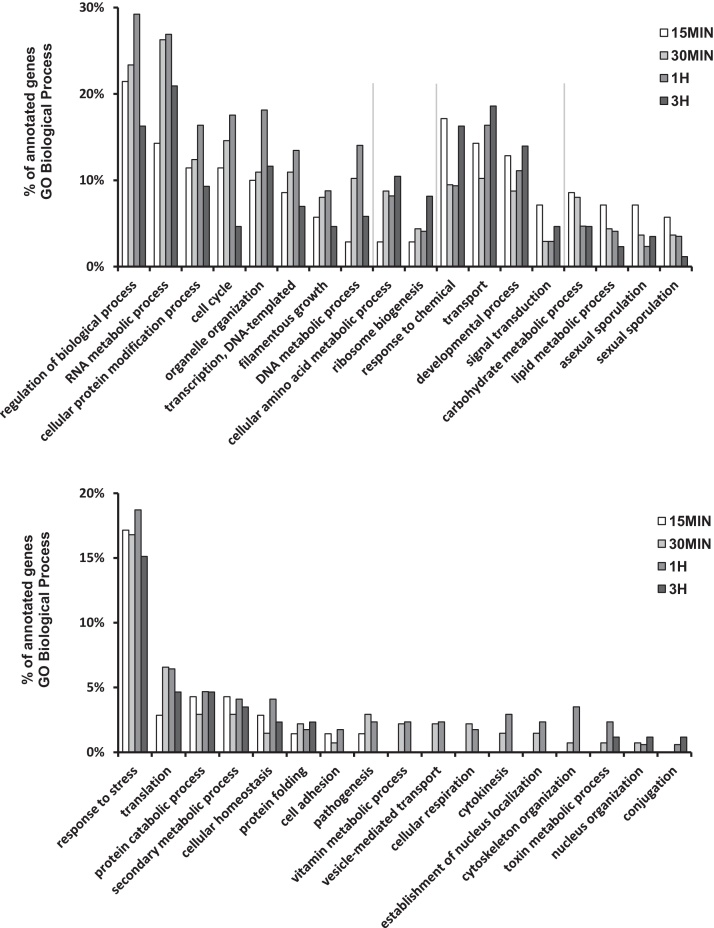
Gene Ontology classification of differentially expressed genes. Genes differentially expressed (*p* < 0.05) once at any time point were annotated to GO slim terms in the GO Biological Process domain. Bars represent relative frequency of annotation to the single GO slim terms as percentage of annotated genes for each time point.

Genes differentially expressed (*p* < 0.05) at least at one time point were annotated to GO slim terms in the GO Biological Process domain. Between 46.1% and 53.0% of differentially expressed genes had GO Biological Process annotations for each time point investigated (supplemental Table 2). It was found that regulation of biological process was the most frequent annotation after 15 min of platelets exposure. Thereafter, relative proportion of genes annotated to regulation of biological process increased and peaked after 1 h followed by a decline after 3 h. Several GO classifications showed a similar pattern, such as DNA metabolic, RNA metabolic and cellular protein modification processes, transcription, organelle organization, cell cycle and filamentous growth. Annotations to cellular amino acid metabolic process and ribosome biogenesis continued to increase after 3 h (Fig. 3). The inverse pattern was observed for the GO slim terms response to chemical, transport, developmental process and signal transduction, that where most frequently after 15 min and 3 h while the proportion of genes annotated to carbohydrate and lipid metabolic processes as well as sporulation decreased over the course of the experiment (Fig. 3). Annotations to response to stress were found frequently at all time points. Other gene ontology terms were rare and/or their frequency was constant.

### FunCat and KEGG enrichment analyses of differentially expressed genes

52.3% to 60.0% of genes differentially expressed (*p* < 0.05) in *A. fumigatus* at least at one time point had FunCat annotations (supplemental Table 2). Second level FunCat enrichment analysis showed emphasis on RNA modification and RNA processing, both categories significantly enriched and down-regulated at the 15 min time point. Early transriptional responses at the 15 min time point displayed significant increase in the categories oxygen and radical detoxification, catalase reaction, detoxification, translation termination and ligand-dependent nuclear receptor. RNA modification displayed a significant down-regulation at the 30 min and 3 h, too, but an up-regulation at 1 h. Most significantly enriched categories were identified after 1 h of exposure to platelets displaying respiration, electron transport and membrane-associated energy conservation, citrate cycle, mitochondrion and oxygen binding being down-regulated. Lipid, fatty acid and isoprenid metabolism, DNA processing and cell death were significantly up-regulated at this time point, as well as RNA modification and RNA processing. All results of the second level FunCat enrichment are given in Table 1.

Third level FunCat enrichment analysis confirmed that the majority of significantly enriched categories were found after 1 h of platelet treatment, with 14 down- and 17 up-regulated categories. Down-regulated genes at the 1 h time point were mainly involved in electron transport, accessory proteins of electron transport and membrane-associated energy conservation, aerobic and anaerobic respiration as well as homeostasis of cations and ion transport. Up-regulated categories comprised DNA recombination and DNA repair, apoptosis (type I programmed cell death), DNA binding, cytoplasmatic and nuclear protein degradation and mRNA processing. At the earlier time points (15 and 30 min) RNA binding, rRNA modification and RNA transport was commonly decreased. At 30 min electron transport, NAD/NADP binding, Fe/S binding accessory proteins of electron transport, membrane-associated energy conservation, purine metabolism and metabolism of primary metabolic sugar derivatives additionally decreased. Distinct differences could be observed in the up-regulated categories. At 15 min oxygen and radical detoxification, together with translation termination and ligand-dependent nuclear receptors increased. At 30 min increased categories mRNA, tRNA processing, pyrimidine metabolism, receptor binding and DNA synthesis and replication were significantly enriched. At the 3 h time point especially RNA binding, rRNA processing, rRNA synthesis and tRNA processing were significantly encriched categories of down-regulated genes. Up-regulated categories with the most hits at this time point were electron transport and detoxification involving cytochrome P450. Results of the third level FunCat enrichment analysis are summarized in Table 2.

Finally, a KEGG enrichment analysis was performed. Only 12.1–21.5% of differentially expressed genes had KEGG annotations. Significantly enriched categories were mainly associated with metabolic pathways, oxidative phosphorylation and purine metabolism being down-regulated and spliceosome and pyrimidine metabolism being up-regulated. KEGG enrichment analysis data are shown in Table 3.

## Discussion

Human platelets participate in antimicrobial host defence and exhibit immune effector characteristics (Fitzgerald et al., 2006; Klinger and Jelkmann, 2002; Page, 1989). We previously demonstrated that platelets exert antifungal effects against *Aspergillus spp*. by inhibiting germination and hyphal elongation, both of which are of major importance in evolving invasive disease and synergistically enhance effects of antifungal drugs *in vitro* (Perkhofer et al., 2008; Perkhofer et al., 2010; Perkhofer et al., 2013). A platelet-to-conidia ratio of 100:1 was sufficient to significantly attenuate hyphal elongation and *A. fumigatus* viability (Fig. 1). In co-culture platelets immediately interact with hyphae, aggregate and cover the fungus and release serotonin that is stored in the dense granules within 30 min (Perkhofer et al., 2008).

The present *in vitro* study investigated the early transcriptional response of *A. fumigatus* to human platelets in a time course, using genome-wide microarrays. 584 differentially expressed genes were identified and, consistent with our previous data, *A. fumigatus* responded more pronounced at 30–60 min when compared to 15 min and 3 h, where fewer genes were regulated. Gene ontology annotation revealed differentially expressed genes to be associated with regulation of biological processes, oxidative phosphorylation/respiration, stress response, RNA processing and amino acid and nucleotide metabolism. Early down-regulation of RNA modification and RNA processing related processes might reflect attenuation of hyphal elongation and germination implemented by platelets. In hand with this down-regulation, oxidative phosphorylation and energy generating processes (e.g. citrate cycle, pyruvate metabolism) are decreased upon platelet exposure. The oxidative phosphorylation system is the major ATP-generating system in eukaryotic cells but also constitutes the major source of reactive oxygen species (ROS) which induce damage in DNA, proteins and lipids leading to cellular impairment. ROS are also produced by immune cells to antagonize invading pathogens. To minimize ROS induced damage, the fungus probably reduces endogenous ROS production by decreasing respiration but ATP biosynthesis, too. This hypothesis is reinforced by the findings, that most notably genes of the NADH:ubiquinone oxidoreductase complex were decreased. This complex accounts for the major ROS production of the oxidative phosphorylation system. Five genes encoding subunits of NADH-ubiquinone oxidoreductases (Afu6g12790, Afu1g12290, Afu5g04370, Afu5g06540, Afu2g13710) were repressed upon platelet exposure.

In turn an increase in detoxifying mechanisms was observed at the early time points. After 15 min, the highest differential expression was observed for mycelial catalase Cat1 (Afu3g02270) that was approximately 10-fold induced. Cat1 is one of two hyphal catalses being responsible for the detoxification of hydrogen peroxide, a compound, which is also produced by macrophages and neutrophils. Consistently, elevated catalase expression has been observed in *A. fumigatus* challenged with neutrophils (Sugui et al., 2008) and airway epithelial cells (Oosthuizen et al., 2011). Furthermore, *A. fumigatus* catalase was induced during early infection applying a murine model (McDonagh et al., 2008) and in hyphae exposed to voriconazole or amphotericin B (da Silva Ferreira et al., 2006; Gautam et al., 2008). FunCat analysis classified Cat1 in ‘oxygen and radical detoxification’; respective genes of this category were significantly enriched after 15 min of platelets exposure; in addition, genes coding for a homolog of two component signaling cascade, *ssk1* (Afu5g08390) and glutathione S-transferase (GSTs) family protein (Afu4g01440) were enriched. GSTs comprise a family of enzymes that detoxify both reactive species and toxic xenobiotics. Elevated GST has been reported in *A. fumigatus* treated with voriconazole and amphotericin B (da Silva Ferreira et al., 2006; Gautam et al., 2008) as well as in a murine model of invasive aspergillosis (McDonagh et al., 2008).

Superoxide dismutases (SODs) play a major role in the detoxification of ROS via dismutation of superoxide anion to oxygen and hydrogen peroxide, which is subsequently degraded by catalases (Lambou et al., 2010). The cytoplasmatic manganese/iron SOD (Afu1g14550) was significantly induced in *A. fumigatus* exposed to human neutrophils and dendritic cells *in vitro* and was elevated *in vivo* in a murine model of lung infection (McDonagh et al., 2008; Morton et al., 2011; Sugui et al., 2008). Additionally, MnSOD was increased in fungi treated with amphotericin B, probably in response to ROS-generation leading to oxidative damage of cell membranes (Gautam et al., 2008). Exposure to human platelets resulted in significantly reduced MnSOD expression that peaked after 60 min, indicating that platelets induce more hydrogen peroxide than superoxide anions. Expression of ROS and other radical detoxification enzymes like Cat1 and GST may be increased by direct contact of hyphae to platelets. At a later time point (1 h) categories related to DNA damage response, cytoplasmatic and nuclear protein degradation, apoptosis and cell death were significantly increased.

Farnesol, an isoprenoid that inhibits proliferation and induces apoptosis, has been shown to increase mRNA expression of genes encoding apoptosis-inducing factor (AIF) like mitochondrial oxidoreductase and NADH-ubiquinone oxidoreductase in *A. nidulans* (Savoldi et al., 2008). These enzymes have been demonstrated to play a role in farnesol-tolerance and resistance to oxidative stress (Dinamarco et al., 2010; Savoldi et al., 2008). Of note, in our study besides the NADH ubiquinone oxidoreductases mentioned above an AIF-like mitochondrial oxidoreductase (Afu7g02070) was repressed upon exposure to platelets in *A. fumigatus*, indicating increased oxidative stress.

After 1 h of platelets exposure various transcription factors and zinc finger proteins were significantly induced. Additionally, AAA family ATPase (Afu7g06680) was approximately 20-fold induced after 30 min and 1 h. These findings resemble the effect of voriconazole treatment and are indicative for stress response and adaptation to the altered environmental stress conditions (da Silva Ferreira et al., 2006).

We observed down-regulation of spermidine synthase (Afu1g13490) after 30 and 60 min of co-cultivation. Spermidine has been demonstrated to be required for the transitions from germ tube to hyphae (and subsequently to tissue differentiation) and secondary metabolism in *Aspergillus nidulans* (Jin et al., 2002). Spermidine is the substrate of deoxyhypusine synthase (Afu5g01740), an enzyme essential for the post-translational modification of lysine to the unusual amino acid hypusine, uniquely found in eukaryotic translation initiation factor 5A. Interestingly, deoxypusine synthase was significantly repressed by platelets, but in contrast was found to be induced in response to human immature dendritic cells (Morton et al., 2011). Mutation of deoxyhypusine synthase in *Saccharomyces cerevisiae* resulted in a severe growth defect and enhanced sensitivity to compounds affecting cytoplasmic membrane and cell wall integrity (Galvão et al., 2013).

The fungal cell wall protects fungi against a hostile environment and represents a target for the host immune system and antifungal substances (Abad et al., 2010). Hence, the maintenance of cell wall integrity and functionality is crucial for *A. fumigatus*. Exposure to platelets resulted in repression of several genes associated with cell wall integrity and maintenance, namely antigenic cell wall galactomannoprotein (Afu4g00870), cell wall glucanase Utr2 (Afu2g03120), cell wall protein (Afu3g08110) and alpha-1,3-glucan synthase Ags2 (Afu2g11270). Likewise, cell wall integrity/maintenance-related genes were repressed by antifungals e.g. Afu3g08110 by amphotericin B or Ags2 by voriconazole (da Silva Ferreira et al., 2006; Gautam et al., 2008). Conversely, galactomannoprotein and Utr2 were induced during the initiation of fungal infection (McDonagh et al., 2008). Expression of Utr2 as well as Afu3g08110 was elevated in *A. fumigatus* challenged with human airway epithelial cells and human immature dendritic cells, respectively (Morton et al., 2011; Oosthuizen et al., 2011).

Maximum changes of differential gene expression were observed after 30 and 60 min with a relatively high overlap between regulated genes whereas changes were less pronounced after 3 h with a significant overlap between 15 min and 3 h (Fig. 2).

The kinetics of these regulations might be explained by a first rapid response to direct hyphae-platelet contact and a second delayed response to secreted substances released by degranulation of platelets (30–60 min). After 3 h transcriptional response is comparable to that after 15 min and might reflect adaptation to direct hyphae-platelet contact.

Despite the overlap between 15 min and 3 h there are interesting differences in the transcriptional responses, e.g. down-regulation of ABC transporter (Afu5g10510) and ABC multidrug transporter Mdr1 (Afu5g06070). These genes encode for members of the ATP-binding cassette (ABC)-type transporters family which in part function as multidrug efflux pumps in azole drug resistance (Abad et al., 2010). Consistently, both ABC transporters have been found to be induced at a transcriptional level by voriconazole in *A. fumigatus* (da Silva Ferreira et al., 2006).

Only limited reports on the transcriptional response of *Aspergillus* spp. to human cells are available. In a previous study, the interaction of *A. fumigatus* with human neutrophils was investigated; 244 genes up-regulated in conidia but not in hyphae were identified (Sugui et al., 2008). Consistent with our findings, the study of Sugui et al. identified major groups of genes associated with transport, transcription and metabolism and it was concluded that these changes in gene expression indicate a reprogramming of conidial metabolic pathways in response to the new environment. Morton et al. identified 210 differentially expressed genes in *A. fumigatus* conidia exposed to monocyte-derived immature dendritic cells (Morton et al., 2011). 146 up-regulated genes were associated with transport, pathogenesis, RNA processing, ribosome biogenesis and oxidation of fatty acids. 68 down-regulated genes were related to fermentation, metabolism, stress response, transport and energy (TCA cycle). The transcriptional response of *A. fumigatus* to 16HBE14o- human bronchial epithelial cells revealed 150 up-regulated and 33 down-regulated genes (Oosthuizen et al., 2011). Identified genes were involved in vacuolar acidification, siderophore biosynthesis, metallopeptidase and formate dehydrogenase activities. Although the genes reported to be differentially expressed in response to neutrophils and immature dendritic cells were categorized to similar biological processes only 16 and 21 genes were commonly identified when compared to our study. An overlap between our findings and the study on airway epithelial cells was minimal with only seven genes identified in common (Table 4). These differences indicate that the transcriptional responses of *A. fumigatus* to human cells vary widely and strongly depend on the cell type being exposed to; in addition, our findings may also reflect different types of interactions between the fungus and human cells.

Neutrophils are the most abundant type of white blood cells and represent essential key elements of the innate immune system. Dendritic cells are the most important antigen presenting cells and act as messengers between the innate and the adaptive immune system. Both cells have been shown to phagocytose *A. fumigatus* conidia (Morton et al., 2011; Sugui et al., 2008), whereas platelets were unable to internalize conidia but adhere to and cover the fungus (Perkhofer et al., 2008). In contrast to platelets, human bronchial epithelial cells have been shown to internalize *A. fumigatus* conidia (Gomez et al., 2010; Oosthuizen et al., 2011).

Previous studies co-cultured conidia with human cells whereas we challenged *A. fumigatus* hyphae with platelets. Moreover, differences in experimental settings like culture conditions, adherent cells vs. cells in suspension and different mircoarray platforms, are likely to have contributed to the small overlap.

However, despite different cell types and experimental settings, 39 genes were identified to be affected in the present study as well as in one of the previous studies (Table 4).

In conclusion, we demonstrated that *A. fumigatus* displayed a specific transcriptional response to exposure to platelets. Detoxification mechanisms were induced at early time points, while respiration and associated processes were down-regulated over the course of time. 1 h platelet exposure induced apoptosis- and cell death-related genes in *A. fumigatus*. This might reflect the potential antifungal capacities of substances released by human platelets. Our data are in accordance with previous studies, showing a synergistic effect of platelets and antifungal drugs (Perkhofer et al., 2010; Perkhofer et al., 2013) suggesting that platelets play a key role in antifungal host defence.

## Figures and Tables

**Table 1:** Second level FunCat Enrichment Analysis* of genes differentially expressed.

**FunCat ID**	**Category**	**15** **min**		**30** **min**		**1** **h**		**3** **h**	
		**Down hits (*****p*****-value)**	**Up hits (*****p*****-value)**	**Down hits (*****p*****-value)**	**Up hits (*****p*****-value)**	**Down hits (*****p*****-value)**	**Up hits (*****p*****-value)**	**Down hits (*****p*****-value)**	**Up hits (*****p*****-value)**
11.06	RNA modification	3 (6.56 × 10^−3^)		3 (4.03 × 10^−2^)			6 (7.10 × 10^−3^)	3 (1.60 × 10^−2^)	
11.04	RNA processing	6 (3.72 × 10^−2^)			18 (3.51 × 10^−4^)		25 (1.16 × 10^−4^)	12 (1.18 × 10^−4^)	
32.07	Detoxification		6 (3.10 × 10^−2^)						
02.13	Respiration			6 (9.29 × 10^−3^)		11 (3.01 × 10^−6^)			
42.32	Flagellum			1 (1.17 × 10^−2^)		1 (1.26 × 10^−2^)			
16.21	Complex cofactor/cosubstrate/vitamine binding			11 (1.72 × 10^−2^)		14 (1.02 × 10^−3^)			
02.11	Electron transport and membrane-associated energy conservation			5 (2.18 × 10^−2^)		9 (4.80 × 10^−5^)			
20.09	Transport routes			6 (2.90 × 10^−2^)	10 (2.01 × 10^−2^)		19 (3.61 × 10^−2^)		
16.03	Nucleic acid binding			15 (4.29 × 10^−2^)		3 (2.10 × 10^−2^)	33 (3.14 × 10^−3^)	13 (7.60 × 10^−3^)	
16.25	Oxygen binding			1 (4.62 × 10^−2^)		1 (4.95 × 10^−2^)			
01.05	C-compound and carbohydrate metabolism				12 (4.24 × 10^−3^)		13 (2.20 × 10^−6^)		18 (2.37 × 10^−2^)
20.01	Transported compounds (substrates)				12 (5.77 × 10^−3^)	29 (1.13 × 10^−3^)	24 (3.14 × 10^−2^)		
01.01	Amino acid metabolism					17 (2.45 × 10^−4^)			
34.01	Homeostasis					9 (9.29 × 10^−3^)			
16.01	Protein binding					10 (1.49 × 10^−2^)	54 (1.13 × 10^−2^)		6 (3.09 × 10^−2^)
02.10	Tricarboxylic-acid pathway					3 (2.88 × 10^−2^)			
10.01	DNA processing					2 (3.05 × 10^−2^)	28 (1.42 × 10^−3^)		
02.09	Anaplerotic reactions					1 (3.74 × 10^−2^)			
42.16	Mitochondrion					5 (4.11 × 10^−2^)			4 (3.48 × 10^−2^)
16.09	Lipid binding					3 (4.29 × 10^−2^)			
11.02	RNA synthesis					5 (4.76 × 10^−2^)			2 (3.85 × 10^−2^)
01.06	Lipid, fatty acid and isoprenoid metabolism						8 (2.13 × 10^−3^)	1 (2.62 × 10^−2^)	
40.10	Cell death						10 (6.73 × 10^−3^)		
42.01	Cell wall						1 (1.51 × 10^−2^)		
12.07	Translational control						6 (1.98 × 10^−2^)		
16.17	Metal binding						27 (4.34 × 10^−2^)		
01.03	Nucleotide/nucleoside/nucleobase metabolism							9 (2.82 × 10^−3^)	
16.19	Nucleotide/nucleoside/nucleobase binding							14 (4.53 × 10^−3^)	
42.27	Extracellular/secretion proteins								2 (1.50 × 10^−2^)
01.07	Metabolism of vitamins, cofactors and prosthetic groups								6 (4.58 × 10^−2^)

* *p*-value cutoff for enrichment analysis 0.05.

**Table 2:** Third level FunCat Enrichment Analysis* of genes differentially expressed.

**FunCat ID**	**Category**	**15** **min**		**30** **min**		**1** **h**		**3** **h**	
		**Down hits (*****p*****-value)**	**Up hits (*****p*****-value)**	**Down hits (*****p*****-value)**	**Up hits (*****p*****-value)**	**Down hits (*****p*****-value)**	**Up hits (*****p*****-value)**	**Down hits (*****p*****-value)**	**Up hits (*****p*****-value)**
11.06.01	rRNA modification	2 (4.43 × 10^−3^)		2 (1.65 × 10^−2^)					
20.01.21	RNA transport	3 (7.44 × 10^−3^)		3 (4.51 × 10^−2^)					
14.13.04	Lysosomal and vacuolar protein degradation	2 (2.22 × 10^−2^)				3 (1.27 × 10^−2^)			
01.03.16	Polynucleotide degradation	3 (2.70 × 10^−2^)							
16.03.03	RNA binding	5 (3.40 × 10^−2^)		8 (2.67 × 10^−2^)				8 (3.26 × 10^−3^)	
32.07.07	Oxygen and radical detoxification		3 (6.28 × 10^−3^)						
12.04.03	Translation termination		1 (4.10 × 10^−2^)						
30.01.11	Ligand-dependent nuclear receptors		1 (4.10 × 10^−2^)						
16.21.08	Fe/S binding			5 (3.02 × 10^−5^)		5 (4.29 × 10^−6^)			
02.11.05	Accessory proteins of electron transport and membrane-associated energy conservation			5 (2.45 × 10^−4^)		6 (2.97 × 10^−6^)			
11.02.01	rRNA synthesis			5 (1.29 × 10^−3^)				5 (2.47 × 10^−4^)	
02.13.03	Aerobic respiration			5 (3.54 × 10^−3^)		8 (1.24 × 10^−5^)			
02.13.01	Anaerobic respiration			2 (4.64 × 10^−3^)		2 (5.34 × 10^−3^)			
16.21.07	NAD/NADP binding			7 (7.91 × 10^−3^)					
20.01.15	Electron transport			9 (1.25 × 10^−2^)		15 (8.57 × 10^−6^)			6 (4.76 × 10^−2^)
01.03.01	Purin nucleotide/nucleoside/nucleobase metabolism			5 (1.83 × 10^−2^)					
01.20.01	Metabolism of primary metabolic sugar derivatives			2 (3.42 × 10^−2^)					
16.21.05	FAD/FMN binding			4 (4.54 × 10^−2^)		5 (1.46 × 10^−2^)			
11.04.03	mRNA processing (splicing, 5′-, 3′-end processing)				13 (4.56 × 10^−4^)		17 (3.67 × 10^−4^)		
11.04.02	tRNA processing				4 (2.05 × 10^−2^)		7 (9.41 × 10^−4^)	3 (1.29 × 10^−2^)	
01.03.04	Pyrimidine nucleotide/nucleoside/nucleobase metabolism				4 (4.19 × 10^−2^)				
16.01.01	Receptor binding				2 (4.28 × 10^−2^)				
10.01.03	DNA synthesis and replication				6 (4.72 × 10^−2^)		10 (6.14 × 10^−3^)		
01.01.05	Metabolism of urea cycle, creatine and polyamines					4 (1.82 × 10^−3^)			
34.01.01	Homeostasis of cations					8 (1.17 × 10^−2^)			
20.01.01	Ion transport					8 (1.56 × 10^−2^)			
01.20.36	Non-ribosomal peptide synthesis					2 (2.58 × 10^−2^)			
02.45.15	Energy generation (e.g. ATP synthase)					2 (3.08 × 10^−2^)			
16.03.01	DNA binding					1 (4.23 × 10^−2^)	22 (3.16 × 10^−3^)		
01.01.11	Metabolism of the pyruvate family (alanine, isoleucine, leucine, valine) and D-alanine					3 (4.46 × 10^−2^)			
10.01.05	DNA recombination and DNA repair						17 (2.18 × 10^−3^)		
40.10.02	Apoptosis (type I programmed cell death)						10 (3.40 × 10^−3^)		
01.05.02	Sugar, glucoside, polyol and carboxylate metabolism						1 (4.87 × 10^−3^)		
11.06.02	tRNA modification						5 (6.32 × 10^−3^)		
14.07.07	Modification by ubiquitin-related proteins						5 (6.94 × 10^−3^)		
10.01.02	DNA topology						7 (7.87 × 10^−3^)		
01.05.03	Polysaccharide metabolism						1 (2.25 × 10^−2^)		
32.01.09	DNA damage response						8 (2.51 × 10^−2^)		
10.03.04	Nuclear and chromosomal cycle						7 (3.21 × 10^−2^)		
20.09.18	Cellular import						5 (4.21 × 10^−2^)		
16.19.03	ATP binding						24 (4.40 × 10^−2^)	12 (3.04 × 10^−3^)	
01.20.31	Metabolism of secondary products derived from L-lysine, L-arginine and L-histidine						2 (4.48 × 10^−2^)		
14.13.01	Cytoplasmic and nuclear protein degradation						12 (4.60 × 10^−2^)		
11.04.01	rRNA processing							6 (4.11 × 10^−3^)	
01.01.09	Metabolism of the cysteine – aromatic group							5 (2.92 × 10^−2^)	
01.20.15	Metabolism of derivatives of dehydroquinic acid, shikimic acid and chorismic acid							2 (4.25 × 10^−2^)	
20.03.22	Transport ATPases							3 (4.74 × 10^−2^)	
32.07.01	Detoxification involving cytochrome P450								3 (2.18 × 10^−2^)
34.11.01	Photoperception and response								2 (2.34 × 10^−2^)
01.25.09	Extracellular lignin degradation								1 (3.25 × 10^−2^)
01.25.03	Extracellular protein degradation								1 (4.05 × 10^−2^)

* p-value cutoff for enrichment analysis 0.05

**Table 3:** Third KEGG Enrichment Analysis* of genes differentially expressed.

**KEGG number**	**Category**	**15** **min**		**30** **min**		**1** **h**		**3** **h**	
		**Down hits (*****p*****-value)**	**Up hits (*****p*****-value)**	**Down hits (*****p*****-value)**	**Up hits (*****p*****-value)**	**Down hits (*****p*****-value)**	**Up hits (*****p*****-value)**	**Down hits (*****p*****-value)**	**Up hits (*****p*****-value)**
1.1.15	Inositol phosphate metabolism		2 (1.44 × 10^−3^)						3 (3.04 × 10^−4^)
1.3.11	Ether lipid metabolism		1 (2.55 × 10^−2^)						
1.2.1	Oxidative phosphorylation			6 (9.49 × 10^−4^)		8 (4.59 × 10^−5^)			
0.1.1	Metabolic pathways			22 (4.95 × 10^−3^)		24 (1.55 × 10^−2^)	12 (1.81 × 10^−2^)		
1.4.1	Purine metabolism			4 (3.97 × 10^−2^)				5 (1.04 × 10^−3^)	
2.1.3	Spliceosome				8 (2.88 × 10^−5^)		9 (5.91 × 10^−5^)		
3.1.1	ABC transporters				1 (4.75 × 10^−2^)			1 (3.03 × 10-^2^)	
1.6.1	beta-Alanine metabolism					3 (8.28 × 10^−3^)			
1.5.6	Lysine biosynthesis					2 (1.69 × 10^−2^)			
1.1.10	Pyruvate metabolism					3 (3.72 × 10^−2^)			
1.8.5	Pantothenate and CoA biosynthesis					2 (4.33 × 10^−2^)			
2.4.5	Homologous recombination						3 (5.79 × 10^−3^)		
2.4.1	DNA replication						3 (3.62 × 10^−2^)		
1.4.2	Pyrimidine metabolism						4 (4.81 × 10^−2^)	4 (2.95 × 10^−3^)	
2.1.1	RNA polymerase							3 (1.79 × 10^−3^)	
1.5.13	Phenylalanine, tyrosine and tryptophan biosynthesis							2 (2.76 × 10^−2^)	
1.1.6	Galactose metabolism								2 (2.70 × 10^−2^)
1.9.9	Limonene and pinene degradation								3 (4.51 × 10^−2^)

* *p*-value cutoff for enrichment analysis 0.05

**Table 4:** List of *Aspergillus fumigatus* genes commonly identified by our study and by previous studies.

**Locus**	**Protein name**	Incubation with Platelets for*				**Incubation with**		
		**15** **min**	**30** **min**	**1** **h**	**3** **h**	**Neutrophils**^a^	**IDCs**^b^	**EACs**^c^
Afu1g02610	rRNA processing protein	−0.66	**−1.1**	−0.19	−0.02		↑	
Afu1g09570	Hypothetical protein	−0.11	0.68	**1.23**	0.1		↑	
Afu1g13370	Aflatoxin B1-aldehyde reductase GliO-like	0.71	**1.35**	0.45	0.84	↑		
Afu1g14550	Mn superoxide dismutase MnSOD	−0.41	−0.97	**−1.96**	−0.31	↑	↑	
Afu1g16550	Dihydrouridine synthase family protein	−0.99	−0.3	0.56	**−1.59**		↑	
Afu2g02310	Actin cortical patch protein Sur7	−0.42	−0.27	**−2.24**	0.58	↑		
Afu2g03120	Cell wall glucanase (Utr2)	−0.47	**−1.18**	−0.05	−0.69			↑
Afu2g07420	Actin-bundling protein Sac6	0.21	0.54	**2.28**	0.88	↑		
Afu2g07500	Prolidase pepP	−0.62	**−1.53**	**−1.1**	0.38			↑
Afu2g10330	Hypothetical protein	−0.3	**2.42**	**2.8**	−0.15		↑	
Afu2g15960	Nucleotide binding protein Nbp35	0.05	0.64	**1.23**	0.07	↑		
Afu2g16750	Nonsense-mediated mRNA decay protein 3	−0.58	0.56	**1.63**	−0.87		↑	
Afu3g00810	Cholestenol delta-isomerase	**−1.1**	−0.75	−0.46	−0.48	↑		
Afu3g00850	Hypothetical protein	−0.75	**−1.13**	−0.98	−0.71	↑		
Afu3g01260	Acetyltransferase, GNAT family family	0	**1.26**	**1.2**	−0.62		↓	
Afu3g01580	GMC oxidoreductase	−0.09	**3.9**	**3.3**	0.03		↑	
Afu3g02270	Mycelial catalase Cat1	3.41	0.17	−0.31	**3.41**	↑		
Afu3g06070	Histone H1	−0.48	**−1.65**	**−1.45**	0.08	↑		
Afu3g07850	Pheromone maturation dipeptidyl aminopeptidase DapB	**−1.28**	−0.69	**−1.33**	-0.62			↑
Afu3g07910	UDP-glucose 4-epimerase	0.76	0.49	0.21	**1.11**		↑	
Afu3g08110	Cell wall protein	−0.32	−0.84	**−1.63**	−0.07		↑	
Afu3g10770	RTA1 domain protein	0.14	**1.68**	**1.86**	**−1.03**		↑	
Afu4g01140	MFS multidrug transporter	**2.16**	**2.23**	**1.12**	**1.49**		↓	
Afu4g05900	Hypothetical protein	−0.36	0.42	**1.14**	−0.64	↑		
Afu4g10410	Aspartate aminotransferase	0.38	−0.12	−0.17	**1.14**	↑		↑
Afu4g11130	Hypothetical protein	**1.35**	**1.31**	0.67	**2.04**		↑	
Afu4g14250	Hypothetical protein	**1.1**	-0.08	0.3	**2.29**		↓	
Afu5g01740	Deoxyhypusine synthase	−0.42	−0.48	**−1.49**	−0.3		↑	
Afu5g02330	Major allergen and cytotoxin AspF1	−0.36	**−1.34**	**−1.46**	−0.34		↓/↑	
Afu5g03560	Glutamyl-tRNA synthetase	0.36	0.58	**1.33**	0.75			↑
Afu5g05830	CorA family metal ion transporter	−0.83	−0.65	**−1.2**	−0.7			↑
Afu6g03680	Hypothetical protein	**1.5**	0.69	0.2	**3.68**		↑	
Afu6g13150	Hypothetical protein	0.84	**1.32**	0.22	0.85	↑		
Afu6g14090	CFEM domain protein	−0.32	**−2.33**	**−2.85**	−0.01	↑		
Afu7g00580	Hypothetical protein	**1.54**	−0.31	0.9	**2.1**		↑	
Afu7g03830	DNA repair protein Rad7, protein	0.52	**1.99**	**2.31**	0.53		↑	
Afu7g04290	Amino acid permease (Gap1)	**1.44**	0.6	−0.39	**1.57**	↑	↑	
Afu8g05710	MFS sugar transporter Stl1	**1.35**	**3.73**	**3.25**	−0.09	↑	↑	
Afu8g07130	AhpC/TSA family thioredoxin peroxidase	0.16	**-2.02**	**−2.59**	0.27	↑		↑

* Log 2 relative fold change.

a Sugui et al.

b Immature dentritic cells, Morton et al.

c Epithelial airway cells, Oosthuizen et al.; ↑…up-regulation; ↓…down-regulation.
