# Supplementary material for: Differential gene expression in Aspergillus fumigatus induced by human platelets in vitro
Source: Int J Med Microbiol. 2015 May;305(3):327–38. doi: 10.1016/j.ijmm.2015.01.002 (PMC4415150; doi:10.1016/j.ijmm.2015.01.002)
Supplement: Supplementary file 1 [file mmc1.pdf]

Supplemental Table 1. Differentially expressed *Aspergillus fumigatus* genes after incubation with platelets for the times indicated expressed as Log 2 relative fold change.

Locus	Protein name	15 min	30 min	1 h	3 h
Afu1g01410	hypothetical protein	-0.99	-0.95	**-1.18**	-0.67
Afu1g01530	GrpB domain protein	0.44	**1.23**	0.75	-0.09
Afu1g01640	kinesin family protein	**1.18**	0.67	**2.22**	-0.12
Afu1g01680	branched-chain amino acid aminotransferase	0.68	**1.57**	**1.61**	-0.58
Afu1g01940	hypothetical protein	0.35	-0.02	-0.11	**1.03**
Afu1g01950	hypothetical protein	-0.37	-0.22	**1.17**	-0.48
Afu1g02070	cytochrome C1/Cyt1	-0.16	**-1.03**	**-1.11**	-0.10
Afu1g02110	Phe-inhibited DAHP synthase AroG	-0.38	0.05	0.53	**-1.09**
Afu1g02610	rRNA processing protein	-0.66	**-1.10**	-0.19	-0.02
Afu1g02630	rRNA processing protein Pwp1	-0.72	**-1.07**	0.10	**-1.12**
Afu1g02985	hypothetical protein	-0.21	**1.14**	**1.24**	-0.13
Afu1g03500	topoisomerase I	0.70	**1.01**	**1.32**	0.31
Afu1g03800	C6 transcription factor	-0.62	-0.81	-0.91	**-1.15**
Afu1g03950	cytochrome P450 sterol C-22 desaturase, putaitve	-0.61	**-1.44**	**-1.56**	-0.45
Afu1g04430	hypothetical protein	**2.18**	0.26	-0.35	**2.66**
Afu1g04540	NADH-cytochrome b5 reductase	0.69	-0.88	**-1.13**	0.48
Afu1g04860	rhodopsin family protein	0.43	**1.05**	**1.72**	0.26
Afu1g05130	PH domain protein	-0.16	**1.13**	**1.29**	-0.76
Afu1g05210	G-protein complex gamma subunit GpgA	-0.15	**-1.58**	**-1.63**	0.54
Afu1g05310	nucleolar ATPase Kre33	-0.90	**-1.20**	-0.30	-0.43
Afu1g05370	hypothetical protein	-0.30	**1.38**	0.55	-0.35
Afu1g05460	hypothetical protein	0.78	0.70	**1.46**	0.93
Afu1g05600	hypothetical protein	0.96	**1.26**	0.49	0.69
Afu1g05690	hypothetical protein	0.89	0.27	-0.29	**1.07**
Afu1g06100	glutaredoxin Grx1	0.01	0.44	**1.26**	-0.03
Afu1g06280	short-chain dehydrogenase/reductase family protein, putative	**1.39**	0.85	0.84	**1.79**
Afu1g06350	pathogenesis associated protein Cap20	**1.26**	**3.52**	**2.92**	0.19
Afu1g06600	60S ribosome biogenesis protein Rrp14	-0.88	-0.83	0.11	**-1.03**
Afu1g07750	SWIRM domain protein Fun19	-0.48	**1.05**	**1.02**	-0.96
Afu1g08930	WD repeat protein	0.28	0.59	**1.03**	0.50
Afu1g09270	transmembrane glycoprotein	**-1.46**	-0.21	-0.33	-0.40
Afu1g09380	hypothetical protein	0.37	0.28	**1.15**	0.20
Afu1g09510	GPI anchored serine-threonine rich protein	0.38	**-1.14**	**-1.67**	**1.61**
Afu1g09530	hypothetical protein	0.51	**1.10**	0.70	0.74
Afu1g09540	vacuolar transporter chaperon Vtc1	0.43	0.73	0.42	**1.03**
Afu1g09550	dynein light chain (Tctex1)	0.30	-0.47	**-1.12**	0.81
Afu1g09570	hypothetical protein	-0.11	0.68	**1.23**	0.10
Afu1g09590	hypothetical protein	**1.10**	0.94	**2.45**	0.41
Afu1g09660	mitochondrial 2-oxodicarboxylate carrier protein, putative	0.00	-0.98	**-1.12**	-0.01
Afu1g09670	HLH transcription factor (GlcD gamma)	-0.01	**1.41**	**1.49**	-0.77
Afu1g09690	tRNA ligase	0.00	**1.60**	**1.96**	**-1.25**
Afu1g09700	ankyrin repeat protein	-0.70	**1.95**	**2.60**	**-1.01**

Afu1g09710	hypothetical protein	0.43	0.78	**1.58**	-0.42
Afu1g09770	La domain family	**-1.28**	**-1.15**	-0.11	-0.94
Afu1g10190	pre-mRNA splicing factor (Prp31)	-0.02	**1.15**	**1.92**	-0.03
Afu1g10200	cell cycle control protein (Cwf4)	0.14	**1.07**	**1.34**	-0.40
Afu1g10340	integral membrane protein, Mpv17/PMP22 family	0.83	**1.46**	**1.69**	0.73
Afu1g10410	DNA repair protein RAD51	0.40	0.46	**1.12**	0.51
Afu1g10470	hypothetical protein	-0.16	0.35	**1.01**	-0.37
Afu1g10560	GTP binding protein	-0.53	-0.96	0.53	**-1.23**
Afu1g10710	mRNA splicing protein (Prp5)	0.44	**1.77**	**1.72**	0.48
Afu1g10850	ubiquitin-like modifier SUMO	0.67	0.75	**1.53**	**1.36**
Afu1g10860	nuclear pore complex protein Nup107	**-1.11**	-0.59	-0.04	-0.40
Afu1g10990	ribosomal RNA processing protein	0.44	0.35	**1.00**	0.21
Afu1g11000	C6 transcription factor	0.08	**1.21**	**1.02**	-0.52
Afu1g11030	xylitol dehydrogenase	-0.40	**-1.21**	-0.28	-0.66
Afu1g11120	hypothetical protein	0.78	0.78	**1.34**	0.65
Afu1g11200	hypothetical protein	0.47	0.67	**1.34**	-0.11
Afu1g11715	hypothetical protein	**-1.22**	-0.73	-0.41	-0.45
Afu1g11900	PQ loop repeat protein	0.05	**2.43**	**2.07**	-0.43
Afu1g12030	hypothetical protein	0.88	**1.45**	**1.48**	0.38
Afu1g12170	translation elongation factor EF-Tu	-0.44	**-1.37**	**-1.36**	0.16
Afu1g12290	NADH-ubiquinone oxidoreductase 19 kDa subunit	-0.20	**-1.50**	**-1.70**	-0.39
Afu1g12800	isocitrate dehydrogenase, NAD-dependent	-0.10	**-1.39**	**-1.68**	-0.29
Afu1g13040	possible dual specificity protein phosphatase 3	-0.30	**2.04**	**1.81**	-0.70
Afu1g13060	regulator of nonsense transcripts	0.18	0.13	**1.03**	-0.68
Afu1g13090	anthranilate synthase multifunctional protein TrpC, putative	-0.48	-0.14	0.01	**-1.13**
Afu1g13200	hypothetical protein	**-1.16**	-0.57	-0.38	-0.56
Afu1g13220	cytochrome P450 pisatin demethylase	0.90	-0.19	0.01	**1.84**
Afu1g13230	hypothetical protein	0.47	**2.22**	**2.81**	**-1.57**
Afu1g13300	GTP cyclohydrolase II	0.06	**1.34**	0.72	-0.15
Afu1g13310	bZIP transcription factor	0.19	**1.98**	**2.13**	-0.14
Afu1g13370	aflatoxin B1-aldehyde reductase GliO-like	0.71	**1.35**	0.45	0.84
Afu1g13430	hypothetical protein	-0.73	-0.37	**-1.40**	-0.28
Afu1g13460	hypothetical protein	-0.39	0.56	**1.22**	-0.58
Afu1g13490	spermidine synthase	-0.36	**-1.56**	**-1.30**	-0.40
Afu1g13570	small nuclear ribonucleoprotein complex protein Nhp2, putative	-0.26	**-1.22**	-0.24	-0.44
Afu1g13870	alpha-1,2-mannosyltransferase (Alg9)	**-1.30**	-0.26	-0.49	-0.10
Afu1g13900	DNA-directed RNA polymerase I subunit (Rpa190), putative	-0.33	**-1.06**	-0.07	**-1.01**
Afu1g14060	C2H2 finger domain protein	0.32	**1.66**	**2.20**	0.04
Afu1g14120	nuclear segregation protein (Bfr1)	-0.31	**-1.57**	-0.93	0.09
Afu1g14550	Mn superoxide dismutase MnSOD	-0.41	-0.97	**-1.96**	-0.31
Afu1g14570	phosphoribosyl-AMP cyclohydrolase	-0.48	**-1.16**	**-1.14**	-0.57
Afu1g14750	C2H2 transcription factor (Sfp1)	0.31	0.42	**1.02**	0.40
Afu1g15030	SDA1 domain protein	-0.58	**-1.07**	-0.01	-0.85
Afu1g15160	hypothetical protein	0.62	**1.10**	0.88	0.83
Afu1g15220	hypothetical protein	-0.07	-0.79	**-1.20**	-0.17

Afu1g15300	choline transport protein	-0.84	-0.88	-0.55	**-1.07**
Afu1g15830	hypothetical protein	**1.12**	**1.22**	**1.52**	**1.25**
Afu1g16280	mitochondrial F1F0-ATP synthase g subunit	0.16	-0.99	**-1.40**	0.32
Afu1g16450	hypothetical protein	-0.71	-0.65	-0.19	**-1.66**
Afu1g16550	dihydrouridine synthase family protein	-0.99	-0.30	0.56	**-1.59**
Afu1g16564	DUF455 domain protein	-0.10	0.97	**2.78**	0.33
Afu1g16570	MIND kinetochore complex component Mtw1	-0.02	**2.07**	**2.40**	-0.18
Afu1g16750	mitochondrial cation transporter	-0.15	-0.27	**-1.02**	-0.50
Afu1g16840	TCTP family protein	0.58	0.17	**2.09**	0.72
Afu1g16940	DEAD/DEAH box helicase	-0.60	0.51	**1.11**	-0.56
Afu1g17270	FRE family ferric-chelate reductase	**-1.00**	-0.94	-0.48	-0.08
Afu1g17400	peptidase S41 family protein	0.27	-0.36	**-1.17**	**1.57**
Afu1g17460	C6 transcription factor	0.41	**1.25**	**1.12**	0.11
Afu1g17670	salicylate hydroxylase	0.19	**-1.07**	-0.25	-0.74
Afu2g00570	GNAT family acetyltransferase	0.53	0.74	**1.02**	**-2.35**
Afu2g00580	hypothetical protein	0.56	**2.97**	**2.97**	0.23
Afu2g00790	hypothetical protein	**1.14**	**2.11**	**1.51**	0.78
Afu2g00930	xylosidase/glycosyl hydrolase	0.65	**1.34**	0.94	0.26
Afu2g00940	hypothetical protein	0.35	**2.92**	**3.15**	-0.13
Afu2g00980	hypothetical protein	0.12	**1.13**	**2.20**	-0.84
Afu2g01110	hypothetical protein	-0.49	**1.63**	**1.79**	-0.43
Afu2g01120	DNA repair protein Ntg1	-0.65	0.93	**1.56**	-0.62
Afu2g01730	hypothetical protein	0.33	0.55	**1.16**	0.20
Afu2g01920	glutaminyl-tRNA synthetase	0.10	0.40	0.57	**-1.03**
Afu2g01980	nuclear protein (Sgd1)	-0.33	**-1.08**	0.19	-0.23
Afu2g02080	C2H2 finger domain protein	0.49	0.69	**1.13**	0.23
Afu2g02290	37S ribosomal protein Rsm22	0.07	0.84	**1.05**	0.10
Afu2g02310	actin cortical patch protein Sur7	-0.42	-0.27	**-2.24**	0.58
Afu2g02490	hypothetical protein	-0.39	0.46	0.44	**-1.99**
Afu2g02530	casein kinase I	**-1.82**	-0.05	-0.27	**-1.63**
Afu2g02590	aspartyl-tRNA synthetase Dps1	-0.28	**1.05**	**1.13**	-0.53
Afu2g02600	hypothetical protein	-0.06	**1.23**	**1.59**	0.15
Afu2g02890	tRNA splicing 2' phosphotransferase 1	0.80	**2.39**	**3.18**	-0.31
Afu2g03120	cell wall glucanase (Utr2)	-0.47	**-1.18**	-0.05	-0.69
Afu2g03260	RNA binding protein (Rbm8A)	-0.98	0.17	-0.11	**-1.32**
Afu2g04470	hypothetical protein	**1.30**	0.75	0.62	**2.31**
Afu2g04550	MFS sugar transporter	0.52	0.70	**1.05**	0.24
Afu2g04600	C6 transcription factor	**-1.10**	-0.39	-0.61	-0.20
Afu2g04700	RNA binding effector protein Scp160	-0.39	**-1.28**	**-1.31**	-0.10
Afu2g05115	hypothetical protein	0.93	**1.19**	0.90	0.44
Afu2g05180	NF-X1 finger and helicase domain protein	0.49	**1.68**	**1.50**	**-1.35**
Afu2g05480	DNA-directed RNA polymerase I 13.1 kDa polypeptide, putative	-0.66	-0.01	-0.38	**-1.29**
Afu2g06110	chromatin remodeling and histone acetyltransferase complexes subunit (Arp4) putative	0.17	0.88	**1.04**	0.01
Afu2g06150	protein disulfide isomerase Pdi1	0.30	0.53	**2.49**	**1.36**

Afu2g06200	hypothetical protein	0.72	0.61	**1.38**	0.28
Afu2g06290	nuclear division Rft1 protein	0.24	**1.06**	**1.13**	0.11
Afu2g06310	ATP-dependent RNA helicase	0.03	0.89	**2.32**	1.00
Afu2g06320	replication protein A 70 kDa DNA-binding subunit	0.71	**1.35**	**3.21**	**1.35**
Afu2g07340	COP9 subunit 3	-0.20	0.24	**1.00**	-0.15
Afu2g07420	actin-bundling protein Sac6	0.21	0.54	**2.28**	0.88
Afu2g07500	prolidase pepP	-0.62	**-1.53**	**-1.10**	0.38
Afu2g07700	hypothetical protein	0.56	0.62	**1.21**	-0.24
Afu2g08300	DnaJ domain protein	**-1.20**	-0.38	0.56	**-1.09**
Afu2g08430	acetyltransferase, CysE/LacA/LpxA/NodL family	-0.15	**-1.07**	**-1.18**	-0.06
Afu2g08550	peptidyl-prolyl cis/trans isomerase	0.25	**1.79**	**1.83**	0.55
Afu2g08590	4'-phosphopantetheinyl transferase NpgA/CfwA	-0.55	**-1.18**	-0.35	-0.17
Afu2g08750	mitochondrial inner membrane nuclease Nuc1	0.25	0.74	**1.17**	-0.21
Afu2g08820	hypothetical protein	**1.30**	0.31	0.11	**1.09**
Afu2g09030	secreted dipeptidyl peptidase DppV	**1.13**	-0.43	**-1.06**	**1.88**
Afu2g09430	hypothetical protein	**2.01**	0.26	-0.65	**2.04**
Afu2g09480	hypothetical protein	0.84	0.56	**1.11**	0.74
Afu2g09510	hypothetical protein	-0.67	0.20	0.34	**-1.53**
Afu2g09710	protein kinase (NpkA)	0.83	0.57	**1.29**	0.23
Afu2g09940	hypothetical protein	-0.34	**-1.10**	-0.17	-0.04
Afu2g10070	carbamoyl-phosphate synthase, large subunit	-0.76	**-1.32**	**-1.46**	-0.44
Afu2g10310	DUF408 domain protein	0.49	**1.21**	**1.48**	-0.36
Afu2g10330	hypothetical protein	-0.30	**2.42**	**2.80**	-0.15
Afu2g10420	branched-chain amino acid aminotransferase, cytosolic	-0.47	-0.65	**-1.47**	**-1.53**
Afu2g10640	SAGA complex subunit (Ada2)	0.28	0.45	**1.15**	0.42
Afu2g10830	hypothetical protein	0.36	0.40	0.82	**1.00**
Afu2g11020	triosephosphate isomerase	**1.16**	-0.56	-0.26	**1.51**
Afu2g11040	U-box domain protein	**-1.14**	0.61	-0.01	**-1.94**
Afu2g11270	alpha-1,3-glucan synthase Ags2	**-1.04**	-0.47	-0.40	-0.50
Afu2g11450	rRNA processing protein Rrp8	-0.02	0.06	**1.22**	0.05
Afu2g11510	nucleolar GTP-binding protein (Nog1)	**-1.23**	**-1.29**	0.14	**-1.41**
Afu2g11730	Protein kinase domain-containing protein	**-1.30**	**-1.33**	-0.10	-0.60
Afu2g12470	acetylornithine aminotransferase	-0.34	**1.13**	**1.10**	-0.63
Afu2g12540	hypothetical protein	0.86	0.70	0.54	**1.14**
Afu2g12590	Lectin C-type domain protein	0.04	**1.26**	**1.08**	0.12
Afu2g12630	allergenic cerato-platanin Asp F13	**1.51**	**1.45**	**1.88**	0.50
Afu2g12680	hypothetical protein	**1.01**	0.45	**1.03**	0.02
Afu2g12770	alpha-L-arabinofuranosidase	-0.59	**-1.09**	-0.21	-0.52
Afu2g13320	mitochondrial membrane fission protein (Fis1)	0.70	0.42	**1.07**	**1.21**
Afu2g13400	membrane protein TMS1	-0.34	**1.43**	**1.21**	-0.88
Afu2g13420	hypothetical protein	-0.63	-0.51	**-1.01**	-0.35
Afu2g13470	pyrimidine 5'-nucleotidase	0.98	**2.00**	**2.47**	**1.11**
Afu2g13710	NADH-ubiquinone oxidoreductase 49 kDa subunit	-0.96	-0.61	0.01	**-1.20**
Afu2g13830	hypothetical protein	-0.28	**1.57**	0.32	0.20
Afu2g13870	mitochondrial carrier protein	-0.08	**1.09**	**1.29**	-0.30
Afu2g13890	annexin ANXC3.2	0.21	-0.06	**1.13**	0.52

Afu2g13930	hypothetical protein	0.16	**1.96**	**2.24**	-0.72
Afu2g13940	hypothetical protein	-0.23	**2.01**	**1.93**	0.00
Afu2g13970	hypothetical protein	0.65	**2.23**	**2.19**	-0.27
Afu2g14090	extragenic suppressor of the bimD6 mutation	0.14	**1.87**	**2.33**	-0.28
Afu2g14370	hypothetical protein	**-1.09**	-0.52	-0.61	-0.68
Afu2g14760	BCS1-like ATPase	**-1.07**	**1.67**	**2.22**	**-1.93**
Afu2g15110	C2H2 finger domain protein	0.67	**3.75**	**4.56**	0.38
Afu2g15165	hypothetical protein	0.20	0.32	**1.10**	0.68
Afu2g15200	hypothetical protein	**2.07**	0.44	-0.12	**1.86**
Afu2g15220	DNA repair protein Pso2/Snm1	-0.44	**1.06**	**1.53**	-0.81
Afu2g15690	hypothetical protein	0.26	0.35	**1.15**	0.55
Afu2g15700	hexokinase family protein XprF	**1.19**	0.62	0.67	0.72
Afu2g15830	TRAF-like signal transducer	-0.09	**1.07**	**1.60**	-0.14
Afu2g15960	nucleotide binding protein Nbp35	0.05	0.64	**1.23**	0.07
Afu2g16060	hypothetical protein	**-1.21**	-0.92	-0.26	-0.15
Afu2g16080	nuclear cohesin complex subunit (Psc3)	**-1.09**	-0.56	-0.99	-0.87
Afu2g16360	DRAP deaminase (Rib2)	0.21	**2.43**	**2.44**	-0.27
Afu2g16370	60S ribosomal protein L32	**1.11**	**1.07**	**2.71**	0.92
Afu2g16390	hypothetical protein	**1.18**	**1.03**	0.69	**1.06**
Afu2g16750	nonsense-mediated mRNA decay protein 3	-0.58	0.56	**1.63**	-0.87
Afu2g16985	integral membrane protein	0.23	0.59	**1.11**	0.11
Afu2g16990	hypothetical protein	**1.17**	**2.68**	**2.54**	**1.12**
Afu2g17240	C2H2 finger domain protein	0.50	**2.18**	**3.30**	-0.03
Afu2g17390	hypothetical protein	-0.22	0.51	**1.38**	-0.14
Afu2g17610	sulfatase domain protein	0.54	**1.20**	**1.55**	**1.20**
Afu2g17840	MFS transporter	-0.24	**-1.95**	**-2.27**	-0.23
Afu3g00560	hypothetical protein	-0.24	**1.11**	0.54	0.05
Afu3g00720	DUF500 and UBA/TS-N domain protein	0.28	-0.10	**2.18**	0.38
Afu3g00730	hypothetical protein	0.50	0.06	**1.56**	0.41
Afu3g00810	cholestenol delta-isomerase	**-1.10**	-0.75	-0.46	-0.48
Afu3g00850	hypothetical protein	-0.75	**-1.13**	-0.98	-0.71
Afu3g01070	tyrosinase	-0.76	**-1.57**	**-2.46**	-0.96
Afu3g01130	cell wall protein	-0.58	**-1.04**	-0.89	**-1.48**
Afu3g01150	GPI anchored cell wall protein	**1.29**	-0.19	-0.17	**1.17**
Afu3g01196	hypothetical protein	0.25	0.90	**1.43**	-0.23
Afu3g01260	acetyltransferase, GNAT family family	0.00	**1.26**	**1.20**	-0.62
Afu3g01440	DUF1212 domain membrane protein	0.56	**1.71**	**1.99**	0.51
Afu3g01530	phosphatidylglycerol specific phospholipase	**1.12**	0.36	0.68	**1.02**
Afu3g01580	GMC oxidoreductase	-0.09	**3.90**	**3.30**	0.03
Afu3g02120	short-chain dehydrogenase/reductase family protein, putative	**1.33**	0.79	**1.37**	**1.12**
Afu3g02270	mycelial catalase Cat1	**3.41**	0.17	-0.31	**3.41**
Afu3g03280	FAD binding monooxygenase	0.86	**1.25**	0.40	-0.06
Afu3g03610	hypothetical protein	0.78	**1.00**	**2.31**	**2.65**
Afu3g03750	hypothetical protein	**1.13**	**1.13**	**1.08**	**1.13**
Afu3g04200	N2,N2-dimethylguanosine tRNA methyltransferase	**-1.17**	-0.25	0.01	-0.84
Afu3g05420	mitochondrial tricarboxylate transporter (Ctp), putative	0.44	0.47	**1.17**	**1.03**

Afu3g05830	hypothetical protein	0.82	**1.75**	**1.82**	0.80
Afu3g05860	exosome complex exonuclease Rrp4	**-1.04**	0.02	-0.22	**-1.16**
Afu3g05940	transcription factor Rba50	0.45	**1.99**	**1.61**	0.06
Afu3g06000	sister chromatid cohesion acetyltransferase Eco1, putative	-0.05	0.83	**1.07**	-0.21
Afu3g06070	histone H1	-0.48	**-1.65**	**-1.45**	0.08
Afu3g06260	translation initiation factor SUI1	0.18	**1.93**	**1.76**	-0.36
Afu3g06290	C6 transcription factor	**-1.32**	-0.26	-0.26	-0.40
Afu3g06510	hypothetical protein	-0.11	-0.67	**-1.27**	0.63
Afu3g06770	mRNA splicing protein	-0.08	**2.02**	**2.18**	-0.01
Afu3g07330	essential protein Yae1	**-1.12**	-0.41	0.17	-0.81
Afu3g07600	hypothetical protein	0.00	0.89	**1.86**	0.16
Afu3g07610	hypothetical protein	0.67	0.03	**1.06**	0.16
Afu3g07820	proteasome regulatory particle subunit Rpt1	0.61	0.49	**1.04**	0.96
Afu3g07850	pheromone maturation dipeptidyl aminopeptidase DapB	**-1.28**	-0.69	**-1.33**	-0.62
Afu3g07870	extracellular serine-rich protein	**1.72**	-0.04	**-1.21**	**1.18**
Afu3g07910	UDP-glucose 4-epimerase	0.76	0.49	0.21	**1.11**
Afu3g08040	polymerase (RNA) II (DNA directed) polypeptide D	0.43	**1.19**	**1.49**	0.64
Afu3g08090	60S ribosomal protein Nsa2	-0.85	-0.67	0.58	**-1.11**
Afu3g08110	cell wall protein	-0.32	-0.84	**-1.63**	-0.07
Afu3g08350	protein transport membrane glycoprotein Sec20	0.18	0.98	**1.45**	0.12
Afu3g08380	inorganic diphosphatase	0.52	0.43	**2.19**	**2.12**
Afu3g08390	tRNA dihydrouridine synthase (Smm1)	-0.53	**1.04**	**1.46**	-0.79
Afu3g09020	DNA primase subunit Pri1	0.45	0.17	**1.34**	-0.01
Afu3g09580	hypothetical protein	-0.15	**-1.13**	-0.58	-0.71
Afu3g09770	RNA-binding protein (Nab3)	**-1.39**	-0.40	-0.13	-0.59
Afu3g09840	hypothetical protein	0.89	0.75	**1.02**	0.35
Afu3g09920	hypothetical protein	**1.24**	**1.14**	1.00	0.49
Afu3g10110	electron transfer flavoprotein-ubiquinone oxidoreductase	0.03	0.65	**1.05**	-0.06
Afu3g10320	RING finger domain protein	0.12	**1.21**	**1.30**	-0.66
Afu3g10440	hypothetical protein	0.51	**1.58**	0.79	0.28
Afu3g10770	RTA1 domain protein	0.14	**1.68**	**1.86**	**-1.03**
Afu3g10800	eukaryotic translation initiation factor 3 subunit CLU1/TIF31	-0.69	**-2.00**	**-1.58**	-0.21
Afu3g10890	ATP dependent RNA helicase (Dbp8)	-0.94	-0.75	0.79	**-1.22**
Afu3g11080	MAP kinase kinase kinase (Bck1)	**-1.43**	**-1.03**	-0.49	-0.91
Afu3g11220	ribonuclease T2 family	**-1.37**	**1.63**	0.51	**-1.07**
Afu3g11310	hypothetical protein	-0.32	-0.46	0.72	**-1.31**
Afu3g11360	eukaryotic translation initiation factor 3 subunit EifCe	0.36	0.22	**1.11**	0.47
Afu3g11630	hypothetical protein	0.25	**1.43**	0.60	-0.01
Afu3g12050	F-box domain protein	0.03	**1.30**	0.87	-0.06
Afu3g12060	Methionine synthase, vitamin-B12 independent	**1.02**	**1.12**	0.31	0.45
Afu3g12320	lipase/serine esterase	0.19	**1.77**	**1.75**	0.09
Afu3g12460	hypothetical protein	**1.04**	0.22	0.38	0.40
Afu3g12490	protein arginine methyltransferase RmtB	-0.55	**-1.36**	-0.18	-0.63
Afu3g12680	hypothetical protein	-0.10	-0.75	-0.57	**-1.20**
Afu3g12710	hypothetical protein	**-1.13**	**-1.24**	-0.44	-0.71
Afu3g12840	Signal peptidase I	-0.53	0.85	**1.31**	**-1.08**

Afu3g12900	MFS transporter	**1.38**	0.17	0.00	**2.25**
Afu3g13110	extracellular serine-threonine rich protein	**1.04**	**-1.18**	**-1.02**	**1.65**
Afu3g13180	hypothetical protein	0.10	**1.48**	**1.99**	-0.27
Afu3g13280	DEAD/DEAH box helicase (Sbp4)	-0.83	**-1.54**	-0.45	**-1.09**
Afu3g13510	flavin containing amine oxidase	0.21	**1.81**	**1.26**	0.49
Afu3g13700	transferase family protein	0.40	**-1.05**	**-1.17**	**1.02**
Afu3g14120	hypothetical protein	1.00	**1.06**	0.73	**1.14**
Afu3g14150	F-box domain protein	0.85	**2.47**	**2.58**	-0.21
Afu3g14590	copper amine oxidase	0.14	-0.99	**-1.44**	-0.22
Afu3g15080	hypothetical protein	-0.38	**2.39**	**3.36**	-0.53
Afu4g00160	short-chain dehydrogenase	**-1.55**	-0.70	**-1.10**	-0.64
Afu4g00200	F-box domain protein	-0.24	**1.68**	**1.80**	**-1.12**
Afu4g00870	antigenic cell wall galactomannoprotein	-0.80	-0.35	-0.46	**-1.02**
Afu4g00970	IMP-specific 5'-nucleotidase (Isn1)	-0.11	-0.22	**2.18**	-0.10
Afu4g00980	hypothetical protein	**-1.20**	0.86	**1.36**	**-1.72**
Afu4g01140	MFS multidrug transporter	**2.16**	**2.23**	**1.12**	**1.49**
Afu4g01210	hypothetical protein	0.67	0.79	**1.61**	-0.09
Afu4g01420	spore wall assembly protein Dit1	**1.20**	-0.28	-0.65	**1.46**
Afu4g01440	glutathione S-transferase family protein	**1.09**	0.34	0.66	0.43
Afu4g02900	sensor histidine kinase/response regulator	-0.74	-0.68	**-1.03**	-0.25
Afu4g02990	vesicle-mediated transport protein (Imh1)	0.51	0.94	**1.23**	0.24
Afu4g03120	mitochondrial cytochrome b2	0.73	-0.68	**-1.19**	**1.63**
Afu4g03170	hypothetical protein	-0.89	**3.24**	**2.95**	-0.52
Afu4g03240	cell wall serine-threonine-rich galactomannoprotein Mp1	**1.76**	**1.09**	0.03	**1.55**
Afu4g03320	membrane bound cation transporter	0.25	0.24	**1.45**	0.91
Afu4g03400	hypothetical protein	0.92	-0.30	0.09	**2.19**
Afu4g03490	tripeptidyl-peptidase (TppA)	-0.57	**-2.01**	**-1.78**	-0.03
Afu4g03650	ribosome associated DnaJ chaperone Zuotin	-0.13	0.48	**1.06**	-0.46
Afu4g03770	chlorohydrolase family protein	-0.78	**1.57**	**1.64**	**-1.39**
Afu4g03820	hypothetical protein	0.52	0.99	**1.09**	0.71
Afu4g03880	60S ribosomal protein L7	-0.89	**-1.17**	**-1.33**	0.09
Afu4g03940	ferric-chelate reductase	-0.66	-0.92	**-1.32**	-0.18
Afu4g04480	hypothetical protein	**-1.48**	-0.77	-0.60	-0.74
Afu4g05870	oxidoreductase, short-chain dehydrogenase/reductase family	0.86	**2.14**	0.99	0.87
Afu4g05900	hypothetical protein	-0.36	0.42	**1.14**	-0.64
Afu4g06010	peptide alpha-N-acetyltransferase Nat2	-0.22	**-1.15**	**-1.21**	-0.28
Afu4g06020	cyclin dependent kinase inhibitor Pho81	-0.07	**-1.14**	-0.40	-0.21
Afu4g06100	hypothetical protein	0.60	**1.29**	**1.28**	0.72
Afu4g06410	hypothetical protein	**1.15**	0.64	0.75	**1.21**
Afu4g06910	outer mitochondrial membrane protein porin	-0.23	-0.63	**-1.26**	0.02
Afu4g06930	methionine aminopeptidase, type II	0.30	0.28	**1.86**	0.64
Afu4g06950	integral ER membrane protein Scs2	-0.21	0.59	**1.66**	0.91
Afu4g07070	hypothetical protein	**1.01**	0.08	0.41	-0.09
Afu4g07430	UBX domain protein	-0.01	**1.95**	**1.63**	-0.08
Afu4g07490	hypothetical protein	-0.38	-0.22	0.22	**-1.46**
Afu4g07500	small nucleolar ribonucleoprotein complex subunit, putative	**-1.09**	**-1.04**	-0.67	-0.95

Afu4g07670	INO80 chromatin remodeling complex (Ies1)	-0.06	0.62	**1.27**	-0.19
Afu4g07680	hypothetical protein	0.20	**1.17**	**1.07**	0.23
Afu4g07710	pyruvate carboxylase	-0.53	-0.95	**-1.36**	-0.53
Afu4g08380	hypothetical protein	**1.29**	0.67	-0.50	**1.44**
Afu4g08420	hypothetical protein	**1.94**	-0.33	0.31	**2.14**
Afu4g08720	lysophospholipase Plb1	-0.06	-0.46	**-1.57**	-0.46
Afu4g08910	hypothetical protein	0.28	**1.71**	**1.58**	-0.26
Afu4g08990	molybdenum cofactor sulfurase protein (HxB)	-0.26	**1.58**	**1.10**	**-1.03**
Afu4g09100	DnaJ domain protein	0.14	**2.09**	**2.69**	0.08
Afu4g09260	hypothetical protein	**2.84**	0.15	-0.13	**3.03**
Afu4g09330	hypothetical protein	-0.05	0.87	**2.04**	0.48
Afu4g09470	cytochrome P450 monooxygenase	-0.24	-0.77	**-1.03**	-0.64
Afu4g09760	hypothetical protein	0.58	**1.95**	**2.24**	0.74
Afu4g09870	hypothetical protein	0.93	0.72	**1.27**	0.57
Afu4g09930	hypothetical protein	**1.12**	**1.24**	**1.31**	**1.03**
Afu4g10260	hypothetical protein	-0.02	0.51	**1.35**	-0.35
Afu4g10410	aspartate aminotransferase	0.38	-0.12	-0.17	**1.14**
Afu4g10430	mitochondrial peptidyl-tRNA hydrolase Pth2	**1.15**	**1.01**	**2.21**	**1.66**
Afu4g10520	CSL family zinc fnger-containing protein	0.42	**2.75**	**3.24**	0.17
Afu4g10850	hypothetical protein	-0.94	**-1.01**	-0.57	**-1.21**
Afu4g11130	hypothetical protein	**1.35**	**1.31**	0.67	**2.04**
Afu4g11320	hypothetical protein	**3.13**	**1.19**	**1.73**	**3.04**
Afu4g11390	ubiquinol-cytochrome c reductase complex 17 kd protein	-0.07	**-1.13**	**-2.00**	0.17
Afu4g11460	aminotransferase, classes I and II	0.03	-0.57	**-1.19**	-0.38
Afu4g11490	hypothetical protein	**1.05**	0.70	0.20	**1.15**
Afu4g11750	hypothetical protein	0.76	0.55	**2.35**	0.88
Afu4g11880	splicing factor 3a subunit 2	-0.02	**1.47**	**1.49**	-0.05
Afu4g11930	formamidopyrimidine-DNA glycosylase	-0.11	-0.48	0.08	**-1.11**
Afu4g11960	iron-sulfur cluster assembly associated protein Nar1, putative	-0.12	**2.43**	**2.21**	-0.58
Afu4g11980	anthranilate phosphoribosyltransferase	0.83	**1.28**	0.98	0.41
Afu4g12030	hypothetical protein	-0.55	**-1.04**	0.24	-0.78
Afu4g12110	TPR domain protein	-0.69	**-1.27**	-0.40	-0.81
Afu4g12170	50S ribosomal protein L2	-0.26	**-1.33**	-0.83	-0.08
Afu4g12350	hypothetical protein	0.19	0.94	**2.21**	0.97
Afu4g12500	hypothetical protein	-0.36	0.58	**1.24**	-0.39
Afu4g12550	hypothetical protein	-0.22	0.02	**1.87**	0.27
Afu4g12720	phosphoesterase	**1.15**	0.74	**1.02**	0.44
Afu4g12940	hypothetical protein	0.94	0.71	**1.42**	0.69
Afu4g13000	lysine-specific histone demethylase Aof2	-0.61	-0.44	**-1.61**	-0.32
Afu4g13360	endo-1,3(4)-beta-glucanase	**1.37**	0.86	0.82	0.55
Afu4g13490	AP-3 adaptor complex subunit mu	0.38	**1.26**	0.55	0.57
Afu4g13860	extracellular salicylate hydroxylase/monooxygenase, putative	0.53	**2.27**	**1.76**	0.26
Afu4g13872	hypothetical protein	-0.50	-0.52	**-1.12**	-0.61
Afu4g14010	short chain dehydrogenase/reductase family protein	-0.29	-0.63	0.10	**-1.07**
Afu4g14030	hypothetical protein	0.81	**1.02**	**1.67**	0.10
Afu4g14060	hypothetical protein	0.86	0.24	-0.04	**1.00**

Afu4g14175	hypothetical protein	**1.26**	**1.94**	**2.65**	**1.86**
Afu4g14250	hypothetical protein	**1.10**	-0.08	0.30	**2.29**
Afu4g14500	hypothetical protein	**1.07**	0.67	-0.50	**1.11**
Afu5g00790	ABC multidrug transporter	0.20	0.69	**1.57**	0.24
Afu5g00800	hypothetical protein	0.35	**2.49**	**2.20**	-0.48
Afu5g00890	integral membrane protein	0.30	0.31	**1.23**	0.27
Afu5g01005	hypothetical protein	**1.28**	0.05	-0.51	**1.71**
Afu5g01010	hypothetical protein	**1.41**	0.07	0.05	**1.57**
Afu5g01242	hypothetical protein	-0.85	**-1.20**	**-1.02**	**-1.42**
Afu5g01300	integral membrane protein	0.91	**1.41**	**1.44**	0.15
Afu5g01740	deoxyhypusine synthase	-0.42	-0.48	**-1.49**	-0.30
Afu5g01770	hypothetical protein	-0.14	**1.08**	**1.23**	-0.62
Afu5g01870	DNA replication factor C subunit Rfc3	-0.58	**-1.06**	-0.57	-0.73
Afu5g01920	GPI anchored protein	**1.15**	**1.98**	**2.69**	0.82
Afu5g02120	F-box domain protein	**1.03**	0.62	0.44	**1.29**
Afu5g02330	major allergen and cytotoxin AspF1	-0.36	**-1.34**	**-1.46**	-0.34
Afu5g02400	catabolite degradation protein	0.73	**1.25**	**1.45**	0.71
Afu5g02720	phosphoribosylformylglycinamidine synthase	-0.65	**-1.56**	-0.72	**-1.37**
Afu5g02760	fatty acid elongase (Gns1)	-0.75	**-1.66**	**-1.65**	0.06
Afu5g03050	transcriptional regulator Cwf13/SkiP	-0.31	**1.14**	**1.24**	-0.85
Afu5g03470	tRNA-guanine transglycosylase family protein	-0.74	**-1.38**	-0.38	-0.87
Afu5g03510	PQ loop repeat protein	-0.47	-0.65	**-1.31**	-0.33
Afu5g03560	glutamyl-tRNA synthetase	0.36	0.58	**1.33**	0.75
Afu5g03700	hypothetical protein	**1.17**	0.59	0.57	0.50
Afu5g03790	ferrooxidoreductase Fet3	0.59	0.43	0.17	**1.11**
Afu5g04040	hypothetical protein	-0.35	0.96	**1.54**	-0.39
Afu5g04050	scramblase family protein	0.30	**1.78**	**1.88**	-0.16
Afu5g04150	hypothetical protein	-0.08	0.93	**1.66**	-0.23
Afu5g04210	ubiquinol-cytochrome C reductase complex core protein 2	-0.31	**-1.52**	**-1.59**	-0.31
Afu5g04370	NADH-ubiquinone oxidoreductase, subunit G	-0.09	**-1.13**	**-1.40**	-0.03
Afu5g04410	hypothetical protein	0.50	**1.24**	**2.08**	-0.80
Afu5g05500	D-3-phosphoglycerate dehydrogenase	0.03	0.24	-0.15	**-1.47**
Afu5g05630	60S ribosomal protein L23	-0.13	-0.13	**1.41**	0.88
Afu5g05730	hypothetical protein	0.59	**1.01**	0.74	0.96
Afu5g05830	CorA family metal ion transporter	-0.83	-0.65	**-1.20**	-0.70
Afu5g05850	hypothetical protein	-0.10	**1.64**	**1.81**	-0.29
Afu5g05930	RNA processing factor 1	**-1.30**	0.10	0.62	**-1.10**
Afu5g05940	peptidyl-tRNA hydrolase	-0.56	**-1.50**	**-1.02**	0.04
Afu5g06060	sulfur metabolism regulator SkpA	0.52	0.89	**1.95**	0.05
Afu5g06070	ABC multidrug transporter Mdr1	0.31	**2.03**	**1.81**	**-1.61**
Afu5g06140	histone acetyltransferase	0.66	**2.09**	**1.99**	0.48
Afu5g06170	hypothetical protein	**-1.49**	0.02	0.51	**-1.63**
Afu5g06400	hypothetical protein	**-1.50**	-0.98	-0.70	**-1.34**
Afu5g06540	NADH-ubiquinone oxidoreductase subunit B17.2	-0.59	-0.85	**-1.16**	-0.17
Afu5g06560	hypothetical protein	-0.40	**-1.01**	-0.63	-0.54
Afu5g06780	carbamoyl-phosphate synthase, small subunit	-0.70	-0.39	-0.41	**-1.03**

Afu5g06915	hypothetical protein	**1.01**	0.87	0.99	0.54
Afu5g07230	carboxyphosphonoenolpyruvate phosphonomutase-like protein	-0.62	-0.63	-0.19	**-1.04**
Afu5g07640	RNA 3'-terminal phosphate cyclase	-0.41	**3.81**	**4.04**	-0.81
Afu5g07850	decapping enzyme Dcp1	0.77	**1.47**	0.65	-0.06
Afu5g07902	hypothetical protein	**-1.05**	-0.84	-0.75	-0.72
Afu5g07960	C2H2 finger and ankyrin domain protein	0.61	**1.52**	**2.90**	0.01
Afu5g08110	origin recognition complex subunit 2	-0.09	**2.93**	**2.89**	-0.63
Afu5g08390	response regulator	**1.21**	**1.17**	0.64	0.76
Afu5g08540	septin AspA	-0.68	**-1.72**	**-1.73**	0.06
Afu5g08650	hypothetical protein	**1.52**	0.62	**1.81**	**1.60**
Afu5g08730	hypothetical protein	0.71	**2.87**	**2.91**	-0.28
Afu5g08750	hypothetical protein	0.17	**1.70**	**1.57**	0.12
Afu5g08800	hypothetical protein	**2.59**	-0.71	-0.80	**2.89**
Afu5g08870	hypothetical protein	0.70	0.52	-0.10	**1.22**
Afu5g08890	homoaconitase LysF	**-1.32**	-0.94	**-1.10**	-0.36
Afu5g08970	oligosaccharyl transferase subunit (beta)	0.42	0.20	0.82	**1.15**
Afu5g09200	ubiquitin conjugating enzyme (UbcC)	0.28	**1.77**	**1.74**	0.04
Afu5g09620	tRNA methyltransferase subunit GCD14	0.10	0.38	**1.04**	0.37
Afu5g10120	NRPS-like enzyme	0.43	**1.47**	**1.78**	0.48
Afu5g10240	hypothetical protein	**1.11**	**1.52**	**1.41**	**1.08**
Afu5g10250	hypothetical protein	0.46	0.68	**1.57**	0.52
Afu5g10290	fructose-bisphosphate aldolase	-0.11	-0.46	**-1.09**	-0.41
Afu5g10470	riboflavin aldehyde-forming enzyme	**-1.21**	-0.51	-0.45	-0.54
Afu5g10510	ABC transporter	-0.24	-0.05	-0.11	**-2.01**
Afu5g10800	G-patch domain protein	-0.45	**1.22**	**1.66**	-0.17
Afu5g11040	pantoate--beta-alanine ligase	-0.36	-0.56	**-1.09**	0.51
Afu5g11630	hypothetical protein	0.88	-0.47	**-1.07**	0.69
Afu5g11670	Leucine carboxyl methyltransferase family	-0.96	**-1.08**	-0.37	-0.77
Afu5g11990	alpha-1,3-mannosyltransferase (Alg3)	-0.80	**-1.25**	-0.10	-0.62
Afu5g12010	serine/threonine protein phosphatase	-0.25	0.98	**1.05**	-0.14
Afu5g12090	hypothetical protein	0.40	0.49	**2.22**	**1.03**
Afu5g12550	N-acetylglucosaminyl-phosphatidylinositol deacetylase, putative	-0.40	-0.62	**-1.23**	-0.33
Afu5g12580	GTP binding protein (GTPBP1)	0.54	**1.93**	**1.97**	0.36
Afu5g12600	DNA excision repair protein (Rad5)	0.63	0.89	**1.21**	0.00
Afu5g12800	50S ribosomal protein L4	-0.96	-0.87	**-1.17**	0.25
Afu5g12890	microfibrillar-associated protein MfaP1	-0.06	0.97	**1.24**	-0.78
Afu5g12895	ssDNA binding protein Ssb3	0.01	**1.19**	**1.08**	-0.10
Afu5g13060	DUF1275 domain protein	**-1.12**	**1.24**	0.34	**-1.23**
Afu5g13220	hypothetical protein	-0.04	**1.56**	**1.36**	0.23
Afu5g13300	aspartic endopeptidase Pep1/aspergillopepsin F	0.30	0.24	-0.24	**1.06**
Afu5g13370	mitochondrial DNA helicase (Pif1)	0.56	0.53	**1.53**	0.17
Afu5g13392	transmembrane GTPase Fzo1	**-1.20**	-0.98	-0.47	-0.84
Afu5g13750	calcium binding protein Caleosin	0.92	0.83	0.48	**1.74**
Afu5g13840	NUDIX domain	-0.26	0.24	**1.20**	-0.14

Afu5g13920	Hsp90 binding co-chaperone (Sba1)	0.31	**1.01**	**1.58**	-0.02
Afu5g14510	beta-lactamase	0.73	0.05	0.00	**1.16**
Afu6g00280	NmrA-like family protein	-0.03	**2.61**	**2.04**	-0.52
Afu6g00310	serine carboxypeptidase (CpdS)	**-1.05**	**-2.93**	**-3.21**	-0.22
Afu6g00520	hypothetical protein	**1.62**	**1.96**	**2.03**	**2.02**
Afu6g00555	hypothetical protein	-0.38	0.60	**1.46**	0.11
Afu6g01900	flavin-binding monooxygenase-like protein	0.96	-0.61	-0.37	**1.06**
Afu6g02200	C2HC5 finger protein	**1.21**	**1.85**	**3.41**	0.53
Afu6g02380	ubiquitin C-terminal hydrolase	-0.86	0.71	**1.17**	**-1.63**
Afu6g02700	structural maintenance of chromosome complex subunit SmcA	0.34	0.29	**1.30**	-0.04
Afu6g02820	metalloreductase	**-1.71**	**-1.27**	**-2.32**	-0.52
Afu6g02830	hypothetical protein	0.03	0.81	**1.38**	-0.29
Afu6g03350	GNAT family N-acetyltransferase	-0.04	0.60	**1.35**	**-2.14**
Afu6g03460	hypothetical protein	**1.18**	-1.00	-0.63	**1.53**
Afu6g03660	onanonoxo-7-onima-8-eninoihtemlysoneda	0.43	-0.47	**-1.15**	0.69
Afu6g03680	hypothetical protein	**1.50**	0.69	0.20	**3.68**
Afu6g04100	Mis6 domain protein	**1.04**	**1.04**	0.37	0.89
Afu6g04180	hypothetical protein	-0.27	**1.76**	**1.31**	-0.67
Afu6g04320	RNA binding protein	0.00	**1.05**	**1.36**	-0.33
Afu6g04750	Hsp70 nucleotide exchange factor (Fes1)	**-1.06**	0.68	0.01	**-1.32**
Afu6g04800	lysine decarboxylase-like protein	**1.08**	**1.65**	**2.78**	**1.06**
Afu6g04970	phosphoserine aminotransferase	0.55	0.86	**1.37**	**-1.02**
Afu6g05040	DNA replication factor C subunit Rfc4	-0.84	**-1.15**	0.44	-0.54
Afu6g06330	adoMet-dependent tRNA methyltransferase (MTase) complex subunit Trm112	0.22	0.55	**1.06**	-0.11
Afu6g06750	14-3-3 family protein	0.19	**-1.10**	-0.19	-0.49
Afu6g06990	von Willebrand and RING finger domain protein	0.72	0.76	0.08	**1.00**
Afu6g07000	hypothetical protein	**2.13**	**2.51**	**1.25**	**3.59**
Afu6g07020	SAM and PH domain protein (Boi1)	**1.78**	-0.54	0.01	**1.74**
Afu6g07100	diphthamide biosynthesis protein Dph2	-0.19	0.71	**1.15**	-0.37
Afu6g07120	nuclear movement protein NudC	0.44	**1.34**	**1.45**	0.11
Afu6g07200	hypothetical protein	**1.70**	0.06	-0.30	**2.71**
Afu6g07370	hypothetical protein	**2.55**	0.15	-0.51	**2.79**
Afu6g07390	isocitrate dehydrogenase LysB	0.02	**-1.67**	**-1.24**	0.23
Afu6g07410	hypothetical protein	**-1.22**	-0.51	-0.46	**-1.04**
Afu6g07430	pyruvate kinase	**-1.65**	**-1.48**	**-1.20**	-0.98
Afu6g07590	hypothetical protein	-0.80	0.86	**1.26**	**-1.01**
Afu6g07700	chromosomal organization and DNA repair protein Mms21, putative	0.57	**1.34**	**1.98**	0.26
Afu6g07970	calpain-like protein	0.54	0.58	**1.46**	0.67
Afu6g08560	NRPS-like enzyme	-0.26	-0.44	**-1.04**	-0.62
Afu6g08570	DNA damage repair protein Mus42	0.69	**1.02**	**1.05**	0.28
Afu6g08790	C6 transcription factor (PrnA)	**-1.16**	-0.51	-0.55	-0.62
Afu6g08860	sugar isomerase, KpsF/GutQ	0.60	**1.13**	**1.31**	-0.41
Afu6g09315	hypothetical protein	0.57	0.56	**-1.22**	0.32

Afu6g10280	hypothetical protein	0.43	**1.83**	**2.13**	0.76
Afu6g10530	transcriptional elongation regulator Elc1/Elongin C, putative	0.45	**1.92**	**1.78**	-0.62
Afu6g10790	MFS amine transporter	-0.10	0.91	0.69	**-1.06**
Afu6g10840	hypothetical protein	-0.09	**1.28**	**1.29**	-0.25
Afu6g10890	actin cytoskeleton organization protein App1	0.76	**1.21**	**1.76**	0.44
Afu6g10940	hypothetical protein	-0.18	0.37	**1.06**	-0.14
Afu6g11070	DEAD box RNA helicase (Hca4)	-0.30	-0.69	0.11	**-1.43**
Afu6g11130	possible replication factor-a protein	0.37	0.69	**1.08**	0.20
Afu6g11160	isopentenyl-diphosphate delta-isomerase	-0.06	0.14	**2.41**	0.43
Afu6g11170	C2H2 finger domain protein (Ezf)	0.35	**1.09**	**1.31**	0.53
Afu6g11560	integral membrane protein	-0.01	**2.23**	**1.98**	-0.43
Afu6g11720	hypothetical protein	-0.58	**-1.21**	-0.14	**-1.28**
Afu6g12145	hypothetical protein	**-1.14**	-0.17	-0.51	-0.76
Afu6g12320	hypothetical protein	-0.76	0.38	0.83	**-1.39**
Afu6g12330	WD domain protein	-0.09	**1.09**	**1.81**	-0.23
Afu6g12540	RING finger protein	0.18	**1.70**	**2.19**	0.05
Afu6g12650	hypothetical protein	0.51	**2.02**	**2.39**	0.16
Afu6g12790	NADH-ubiquinone oxidoreductase 39 kDa subunit	-0.25	**-1.98**	**-2.43**	-0.22
Afu6g12900	mitochondrial carrier protein	0.25	**1.36**	**1.00**	-0.54
Afu6g13100	magnesium dependent phosphatase	0.18	0.92	**1.25**	0.07
Afu6g13150	hypothetical protein	0.84	**1.32**	0.22	0.85
Afu6g13210	oxidoreductase, short-chain dehydrogenase/reductase family	0.72	0.35	-0.46	**1.07**
Afu6g13310	26S proteasome non-ATPase regulatory subunit Nas2, putative	-0.22	0.97	**1.46**	0.19
Afu6g13346	hypothetical protein	0.34	0.31	-0.17	**1.20**
Afu6g13640	hypothetical protein	0.20	**2.61**	**1.94**	0.00
Afu6g13750	ferric-chelate reductase	-0.66	-0.63	**-1.06**	0.33
Afu6g14090	CFEM domain protein	-0.32	**-2.33**	**-2.85**	-0.01
Afu6g14270	hypothetical protein	0.45	**1.58**	0.84	0.26
Afu6g14360	cytochrome P450 monooxygenase	0.89	0.36	-0.10	**1.37**
Afu6g14490	beta-glucosidase	**-1.29**	-0.50	-0.70	-0.52
Afu7g00180	NAD dependent epimerase/dehydratase	0.20	0.59	**2.04**	0.29
Afu7g00310	hypothetical protein	-0.66	**-1.04**	-0.91	**-1.46**
Afu7g00440	GABA permease	0.89	-0.03	-0.26	**1.04**
Afu7g00580	hypothetical protein	**1.54**	-0.31	0.90	**2.10**
Afu7g00820	hypothetical protein	**1.43**	**1.12**	0.83	**1.54**
Afu7g01240	phytase	0.48	-0.79	**-1.23**	**1.19**
Afu7g01620	hypothetical protein	-0.09	**1.32**	**1.37**	0.20
Afu7g01720	3-hydroxymethyl-3-methylglutaryl-Coenzyme A lyase	0.74	**1.10**	0.71	0.74
Afu7g01920	DNA-dependent RNA polymerase II RPB140	0.37	0.99	**1.40**	0.30
Afu7g01930	GTP-binding protein EsdC	**1.83**	-0.51	-0.28	**2.37**
Afu7g01940	nuclear pore complex subunit Nup85	-0.03	0.40	**1.24**	0.23
Afu7g02050	arrestin (or S-antigen), N-terminal domain protein	1.00	**1.25**	**1.27**	**1.80**
Afu7g02070	AIF-like mitochondrial oxidoreductase (Nfrl)	0.37	**-2.23**	**-2.20**	0.26
Afu7g02080	RNA polymerase I subunit Rpa43	**-1.07**	**-1.65**	-0.39	**-1.14**
Afu7g02230	mRNA binding post-transcriptional regulator (Csx1), putative	-0.73	**-1.28**	-0.44	**-1.30**

Afu7g03830	DNA repair protein Rad7, protein	0.52	**1.99**	**2.31**	0.53
Afu7g03970	hypothetical protein	-0.46	**-1.31**	**-1.38**	0.17
Afu7g03980	PCI domain protein	-0.60	-0.43	-0.16	**-1.03**
Afu7g04040	hexokinase	0.29	0.14	-0.08	**1.00**
Afu7g04140	L-galactose dehydrogenase (L-GalDH)	0.66	**1.15**	**1.04**	0.51
Afu7g04290	amino acid permease (Gap1)	**1.44**	0.60	-0.39	**1.57**
Afu7g04500	ATP phosphoribosyltransferase His1	-0.65	**-1.69**	**-1.61**	-0.69
Afu7g04530	alcohol dehydrogenase, zinc-containing	**2.19**	**1.01**	0.83	0.29
Afu7g04560	hypothetical protein	-0.21	-0.55	**-1.07**	0.16
Afu7g04870	glutamine-serine-proline rich protein	0.35	0.90	**1.74**	0.21
Afu7g05370	septin AspB	-0.27	-0.61	**-1.32**	0.13
Afu7g05420	mitochondrial intermembrane space protein Mia40	-0.08	**-1.03**	**-1.58**	0.12
Afu7g05730	hypothetical protein	0.43	**1.05**	0.78	-0.02
Afu7g06080	ubiE/COQ5 methyltransferase	**1.49**	-0.13	0.07	**1.16**
Afu7g06270	cyanamide hydratase	0.76	-0.22	0.00	**1.04**
Afu7g06440	F-box domain protein	0.01	-0.36	-0.27	**1.00**
Afu7g06630	cytochrome P450 monooxygenase	**1.18**	0.43	0.40	**1.08**
Afu7g06675	COG3602 family protein	0.30	**1.15**	**1.19**	0.15
Afu7g06680	AAA family ATPase	**2.28**	**4.11**	**4.40**	0.07
Afu7g06690	flavin-containing monooxygenase	-0.42	**3.96**	**3.72**	-0.49
Afu8g01310	ferric-chelate reductase (Fre2)	**1.09**	0.67	0.28	0.17
Afu8g01370	CmcJ-like methyltransferase	0.68	0.41	0.49	**1.07**
Afu8g01390	glycosyl hydrolase	0.89	**-1.43**	**-2.07**	0.82
Afu8g01820	hypothetical protein	**-1.26**	-0.74	-0.60	-0.51
Afu8g02060	glycan biosynthesis protein (PigL)	-0.28	-0.75	**-1.04**	-0.50
Afu8g02250	hypothetical protein	-0.26	0.89	**1.35**	-0.25
Afu8g02490	hypothetical protein	-0.30	**1.96**	**2.29**	-0.85
Afu8g02810	ELMO/CED-12 family protein	0.47	**1.02**	**1.09**	0.56
Afu8g02830	UPD-GlcNAc transporter (Mnn2-2)	0.24	-0.73	**-1.43**	-0.01
Afu8g02870	hypothetical protein	0.93	**1.87**	**1.37**	0.34
Afu8g03930	Hsp70 chaperone (HscA)	-0.23	**-1.13**	-0.68	-0.16
Afu8g04030	LMBR1 domain protein	0.19	**1.04**	0.76	0.38
Afu8g04250	pentatricopeptide repeat protein	0.05	**1.02**	**1.51**	-0.52
Afu8g04260	translocation protein (Sec66)	0.41	0.42	**1.99**	0.88
Afu8g04540	C6 transcription factor	-0.43	-0.12	**-1.18**	-0.38
Afu8g05140	oxidoreductin	**1.07**	**1.91**	**1.16**	**1.10**
Afu8g05170	autophagy protein Apg6	0.58	**1.48**	**1.18**	0.56
Afu8g05330	methylenetetrahydrofolate dehydrogenase	-0.59	**-1.38**	-0.91	-0.82
Afu8g05650	hypothetical protein	**1.11**	0.47	**1.67**	**1.17**
Afu8g05710	MFS sugar transporter Stl1	**1.35**	**3.73**	**3.25**	-0.09
Afu8g05720	DUF567 domain protein	0.34	0.23	**1.44**	0.29
Afu8g06070	hypothetical protein	0.10	**-1.18**	-0.09	**1.51**
Afu8g06405	isoflavone reductase family protein	**1.17**	0.83	0.29	**1.15**
Afu8g07090	extracellular proline-serine rich protein	0.64	-0.76	**-1.10**	0.90
Afu8g07130	AhpC/TSA family thioredoxin peroxidase	0.16	**-2.02**	**-2.59**	0.27
