# Supplementary material for: Differential gene expression in Aspergillus fumigatus induced by human platelets in vitro
Source: Int J Med Microbiol. 2015 May;305(3):327–38. doi: 10.1016/j.ijmm.2015.01.002 (PMC4415150; doi:10.1016/j.ijmm.2015.01.002)
Supplement: Supplementary file 2 [file mmc2.pdf]

Supplemental Table 2.

Number of Genes differentially regulated	15 min	30 min	1 h	3 h
total	132	279	371	172
annotated GO Biological Process	70 (53.0%)	137 (49.1%)	171 (46.1%)	86 (50.0%)
annotated FunCat	69 (52.3%)	167 (59.9%)	223 (60.1%)	93 (54.1%)
annotated KEGG	16 (12.1%)	60 (21.5%)	77 (20.8%)	33 (19.2%)
