# Supplementary material for: Differential gene expression in Aspergillus fumigatus induced by human platelets in vitro
Source: Int J Med Microbiol. 2015 May;305(3):327–38. doi: 10.1016/j.ijmm.2015.01.002 (PMC4415150; doi:10.1016/j.ijmm.2015.01.002)
Supplement: Supplementary file 3 [file mmc3.pdf]

A

Locus	Protein name	sense primer sequence 5´-3´	antisense primer sequence 5´-3´
Afu1g17270	FRE family ferric-chelate reductase	AGCATCGTGCTCTGCCTG	AAGAGAGGATCCCGGTGC
Afu2g02530	casein kinase I	CGGCCGTAAGATCGGAAG	TGCTTGGCCTTGACGCTC
Afu2g09030	secreted dipeptidyl peptidase DppV	GGACGCGAAGGTCTCAAC	AACTGGAGGATCCGCTCG
Afu2g11510	nucleolar GTP-binding protein (Nog1)	CGCCTTCTGAAGCTAGGC	TGCCGATCGTGCGCTTTC
Afu2g13710	NADH-ubiquinone oxidoreductase 49 kDa subunit	GGTTTCGAGGAGCGTGAG	AAGGCCAAGGGGAAGGTC
Afu3g02270	mycelial catalase Cat1	CTGGCCCGTGATGTTCAC	ACGGCGTGGATCAGATCG
Afu3g13110	extracellular serine-threonine rich protein	CAGTGACAGCACCGGTTG	TTGGAGGTGGAGAGGTGC
Afu4g01140	MFS multidrug transporter	AGGTGTTCCTGCGTTCGG	AGAAACGCCAGCAGCCAC
Afu4g11800	Alkaline protease alp1	TTCTGTCGTCGCTGCTGG	AGTTGGAGAAGGAGGCGC
Afu5g02330	major allergen and cytotoxin AspF1	CCACAGCCGTGTCTGTTC	CTGTATAGAAGCCGCTTGTC
Afu5g13060	DUF1275 domain protein	CTACAACGCCTGGTCGTG	TCAGCGACTTGAGCCAGC
Afu6g03890	spore-specific catalase CatA	CGAATGACCGCCGAACTG	TTCCGAATGCGCCAGTGC
Afu6g04800	lysine decarboxylase-like protein	CGGCGAGGAAACATGGTC	GTGGAATTCGTGGGCGAG
Afu6g07000	hypothetical protein	GCAACAAGCAACGGACCG	GCCTGGTGTACGTGCATC
Afu6g09740	thioredoxin reductase GliT	GATGTCGCCAAGACCGTC	TCAAGGCGAGCTGCTTGG
Afu7g00250	beta tubulin	CTGTCGGTCCACCAGTTG	GGTTGAGATCGCCGTACG
Afu7g02230	mRNA binding post-transcriptional regulator (Csx1), putative	CATGCCCAACACCAACCG	TCGTTGACTTCAGGGCCG

B

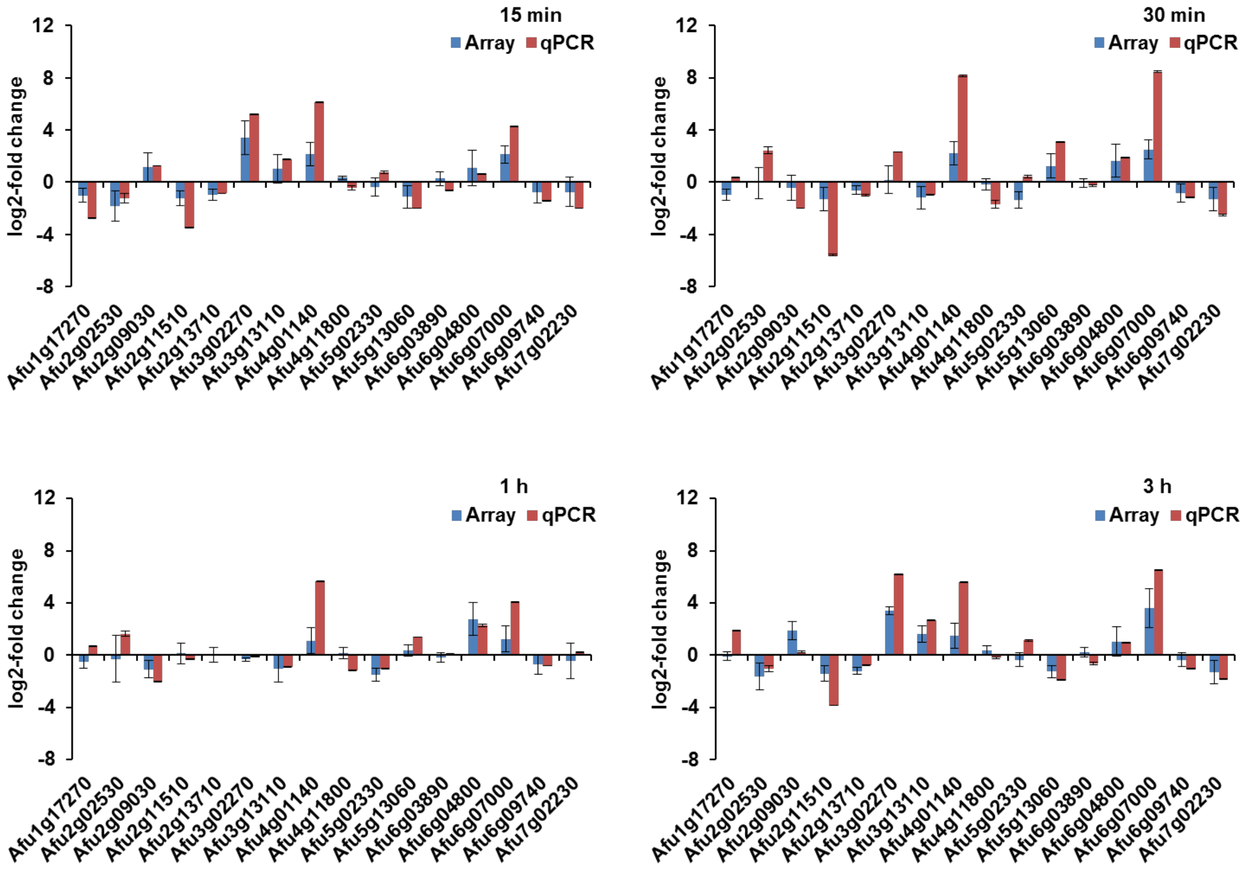

Supplemental Figure 1: RT-qPCR Validation of microarray results. (A) List of *A. fumigatus* gene identities and primers used for RT-qPCR analysis. (B) Fungal gene expression analysis (log2-fold change) after 15 min, 30 min, 1 h and 3 h of exposure to platelets as determined by microarrays in comparison to RT-qPCR analysis. Error bars indicate SEM.
