# Supplementary material for: Differential gene expression in Aspergillus fumigatus induced by human platelets in vitro
Source: Int J Med Microbiol. 2015 May;305(3):327–38. doi: 10.1016/j.ijmm.2015.01.002 (PMC4415150; doi:10.1016/j.ijmm.2015.01.002)
Supplement: Supplementary file 4 [file mmc4.pdf]

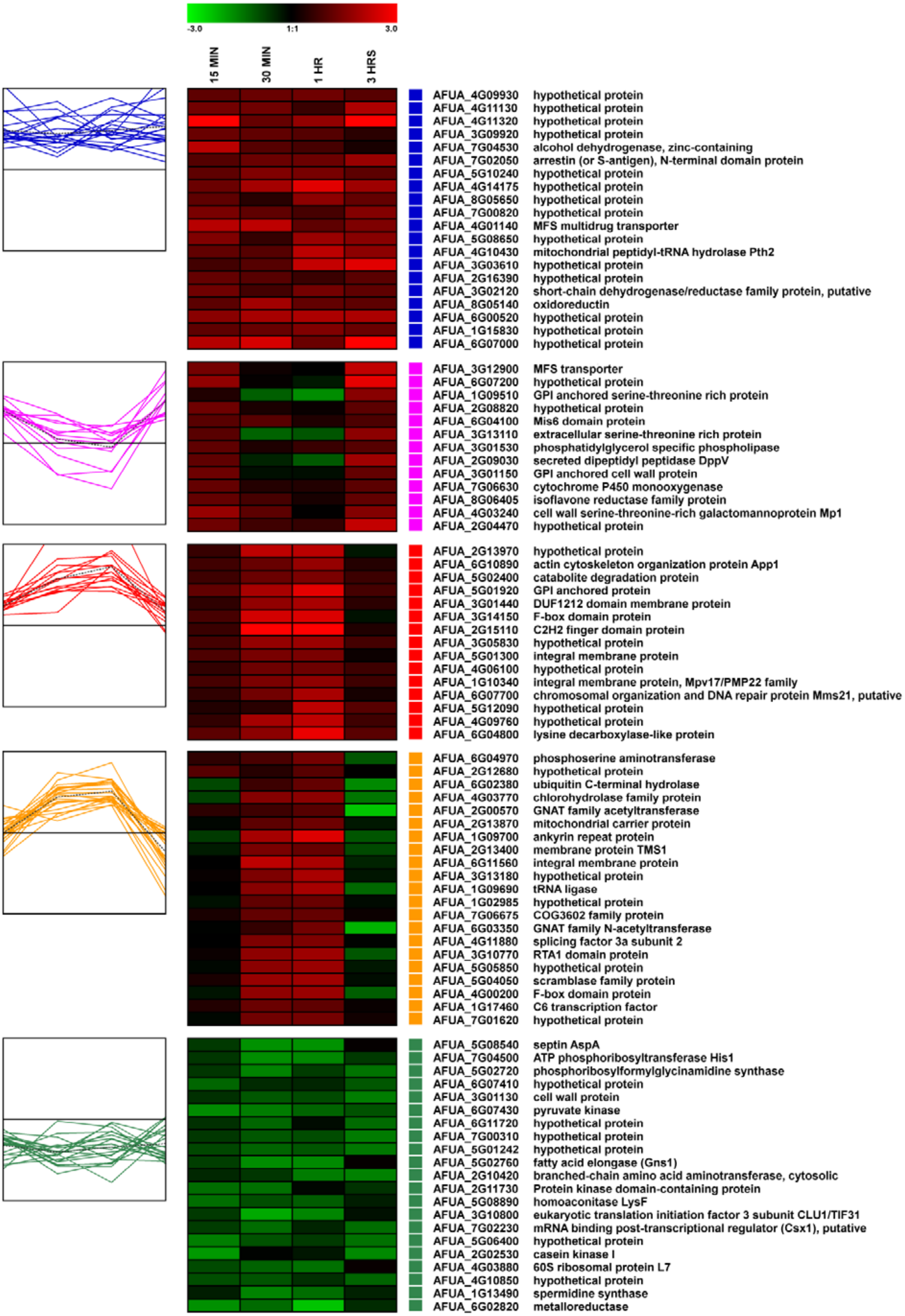

Supplemental Figure 2: K-means clustering of gene expression profiles of genes significantly ($p<0.05$) differentially expressed in *A. fumigatus* at least at two time points after exposure to platelets.
